# The bacterial phosphotransferase system-mediated rifampicin phosphorylation: ancestral links to rifampicin-inactivating enzyme

**DOI:** 10.3389/fmicb.2026.1789656

**Published:** 2026-04-08

**Authors:** Šárka Bobková, Michaela Plechatá, Tomáš Kovaľ, Hana Šanderová, Simona Blažková, Rüveyda Akçin, Zdeněk Kameník, Jan Dohnálek, Libor Krásný, Jana Wiedermannová

**Affiliations:** 1 Laboratory of Microbial Genetics and Gene Expression, Institute of Microbiology of the Czech Academy of Sciences, Prague, Czechia; 2 Laboratory of Antibiotic Resistance and Microbial Metabolomics, Institute of Microbiology of the Czech Academy of Sciences, Prague, Czechia; 3 Laboratory of Structure and Function of Biomolecules, Institute of Biotechnology of the Czech Academy of Sciences, Vestec, Czechia

**Keywords:** ancestral protein, antibiotic resistance, Enzyme I, phosphotransferase system, PTS, rifampicin, rifampicin phosphotransferase

## Abstract

**Introduction:**

The bacterial phosphotransferase system (PTS) transports and phosphorylates sugars. Some PTS proteins share structural motifs with rifampicin phosphotransferases (RPHs), which inactivate rifampicin by phosphorylation. This homology suggests that the PTS may represent an evolutionary ancestor of the multi domain RPHs, though direct biochemical evidence has been lacking.

**Methods:**

*Bacillus subtilis* strains lacking genes encoding PTS proteins were evaluated in growth assays in the absence/presence of rifampicin; liquid chromatography-mass spectrometry was used to monitor the ability of *B. subtilis* PTS proteins to phosphorylate rifampicin; thermophoresis was employed to characterize protein–rifampicin interactions.

**Results:**

Deletion of *B. subtilis ptsH*, *ptsI* genes (encoding PTS proteins: HPr and EI) or *rphT* (encoding RphT-*B. subtilis* RPH) impaired growth in the presence of rifampicin. *In vitro*, the PTS complex (HPr, EI, MtlF, and PckA) phosphorylated rifampicin, with EI alone sufficient for this activity. However, no rifampicin phosphorylation by EI was detected *in vivo*. Heterologous expression of *rphT* then strongly increased rifampicin resistance, while *ptsH/ptsI* expression did not.

**Conclusion:**

This study shows that part of the PTS, protein EI, can phosphorylate rifampicin, supporting its evolutionary link to RPHs. We also establish that RphT, a putative rifampicin phosphotransferase misannotated as phosphoenolpyruvate synthase (Pps), is a *bona fide* rifampicin-modifying enzyme in *B. subtilis.* Finally, we demonstrate that derepressing RphT or its horizontal transfer confers high-level resistance to rifampicin.

## 1 Introduction

Rifampicin is a member of the ansamycin family of antibiotics that exerts bactericidal activity by binding to the β-subunit of bacterial RNA polymerase (RNAP), where it sterically blocks the elongation of the nascent RNA chain (McClure and Cech, 1978; Campbell et al., 2001; Lin et al., 2017). Due to its potent activity against a broad spectrum of pathogens, rifampicin is a cornerstone of therapy for tuberculosis, leprosy, and a variety of acute and chronic infections caused by *Mycobacterium spp., methicillin-resistant Staphylococcus aureus (MRSA), Neisseria meningitidis, and Haemophilus influenzae* (Hardie and Fenn, 2022).

Resistance to rifampicin poses a continuous threat to treatment efficacy. It can arise via mutations in the RNAP β-subunit or through RNAP protection, preventing rifampicin binding, as well as through efflux pumps or enzymatic inactivation by rifampicin ribosylation, glycosylation, or phosphorylation (Quan et al., 1997; Spanogiannopoulos et al., 2012, 2014; Zaw et al., 2018; Hurst-Hess et al., 2022; Surette et al., 2022; Kovaľ et al., 2024; Sudzinová et al., 2026). The latter mechanisms chemically modify rifampicin, thereby rendering the drug inactive.

One such rifampicin-modifying enzyme is the rifampicin phosphotransferase (RPH) found in many environmental and pathogenic bacteria. RPH catalyzes the transfer of a phosphoryl group from ATP to the C-21 hydroxyl of rifampicin. The resulting phosphorylated rifampicin (rif-P) no longer fits into the rifampicin binding pocket in RNAP (Lin et al., 2017).

**Figure 1:**
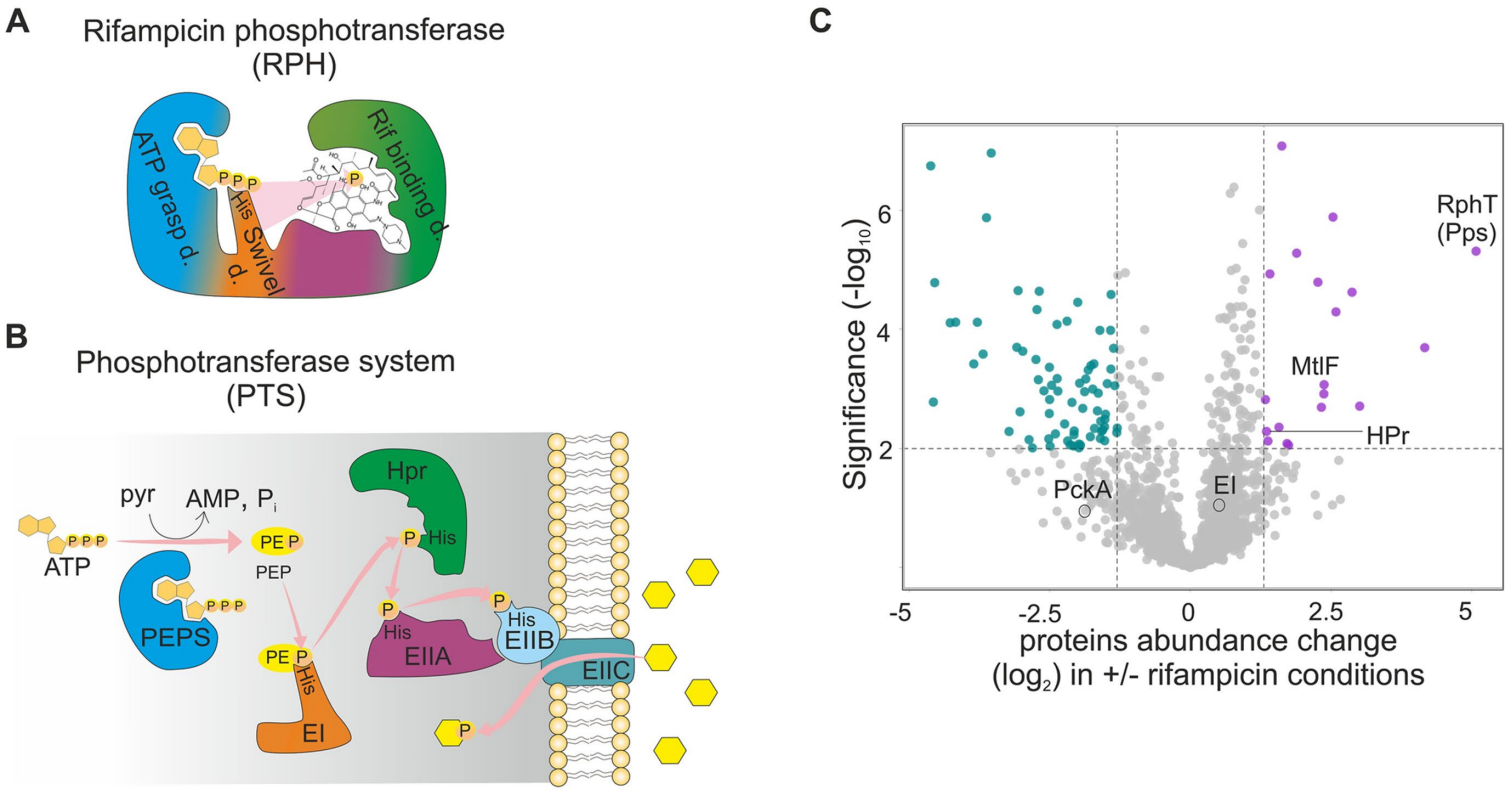
Rifampicin phosphotransferase (RPH) domains possess structural homology with phosphotransferase system (PTS) components. **(A, B)** Schematic representation of rifampicin phosphotransferase (RPH) = RphT in *B. subtilis*. The various colors represent different RPH domains; color-coding in panels **(A)** and **(B)** highlights the structural homology to the respective protein in the PTS cascade depicted in **(B)**. d, domain; Rif, rifampicin. Rifampicin is presented as a chemical formula. **(B)** Scheme of PTS in Gram-positive bacteria. OAA, oxalacetate; PEP, phosphoenolpyruvate; PEPS, phosphoenolpyruvate synthase; EI, Enzyme I of the PTS; HPr, histidine-containing phosphocarrier protein of the PTS; EIIABC, membrane proteins of the PTS system, His refers to histidine residues that are phosphorylated/dephosphorylated during the sugar transport, yellow hexagons represent sugar transported and simultaneously phosphorylated upon cell entry via the PTS cascade. **(C)** Quantitative mass spectrometry proteomic analysis of the *B. subtilis* wild type strain grown without and with sub inhibitory concentrations of rifampicin (0.03 μg/mL) (adapted from Sudzinová et al., 2026). The enrichment is shown with a volcano plot (−log_10_ *p*-value > 2 on the y-axis, protein enrichments > 1.3 on the *x*-axis). Proteins with significantly changed expression are shown as purple (upregulated) or turquoise (downregulated) dots. Only the proteins previously connected to rifampicin resistance and the PTS system proteins relevant for this work are labelled. RphT (Pps), rifampicin phosphotransferase; MtlF, mannitol-specific PTS enzyme IIA (EIIA_mtlF_); HPr, histidine-containing phosphocarrier protein of the PTS; EI, Enzyme I of the PTS; PckA, phosphoenolpyruvate carboxykinase.

3D structure of RPH was solved from *Listeria monocytogenes* (PDB IDs: 5FBS, 5FBT, 5FBU) (Stogios et al., 2016), (PDB IDs: 5HV1, 5HV2, 5HV3, 5HV6) (Qi et al., 2016). It possesses (i) an ATP-grasp domain that binds and hydrolyses ATP, (ii) a His-swivel (phosphate-carrying) domain that relays the phosphate from ATP to the substrate, and (iii) a rifampicin-binding domain, where rifampicin binds and is phosphorylated (Figure 1A) (Stogios et al., 2016). Bioinformatic analyses have revealed that each of these domains shows structural similarity to phosphoenolpyruvate synthases (PEPS) and to distinct components of the bacterial phosphoenol-pyruvate:sugar phosphotransferase system (PTS) (Figures 1A,B) (Stogios et al., 2016).

RPH proteins are often misannotated as phosphoenolpyruvate synthases (PEPs), or pyruvate phosphate dikinases (Stogios et al., 2016) as exemplified in *B. subtilis*, where the respective gene encoding RPH is named *pps* in the Subtiwiki database (Elfmann et al., 2025) and *rph* in UniProt (protein O34309, inferred from homology). Moreover, in *B. subtilis*, *rph* is coincidentally already assigned to a gene encoding RNase PH, a 3′-5′ exoribonuclease (Craven et al., 1992). As our work experimentally shows (see Results) that the *pps* gene indeed encodes an enzyme phosphorylating and deactivating rifampicin, we suggest here (to avoid confusion) to rename the *B. subtilis* *pps* gene to *rphT* (rifampicin phosphoTransferase). In the following text, RPH is used as the general name for rifampicin phosphotransferase, while RphT is used for the same enzyme from *B. subtilis*.

PTS is a cascade of phosphocarrier proteins that links sugar uptake to its simultaneous phosphorylation, effectively coupling transport with the central carbon metabolism. The system is exclusively present in Eubacteria and has long been considered an attractive target for antimicrobial development because it is absent from eukaryotes. In Gram-positive bacteria, it consists of (i) membrane proteins EIIA, EIIB, EIIC, and occasionally EIID, which are specific for one or several sugars or sugar derivatives, and (ii) conserved cytoplasmic components: the histidine-containing phosphocarrier protein HPr, encoded by the *ptsH* gene, and Enzyme I (EI), encoded by the *ptsI* gene. HPr and EI are shared components for all types of transported substrates (Figure 1B).

As mentioned above, the RPH domains share structural homology with four PTS-related proteins: (i) the ATP-grasp domain of RPH is structurally similar to PEP synthases, (ii) the phospho-His swivel of RPH aligns with enzyme I (EI), and (iii) the substrate-binding region of RPH matches HPr/EIIA. Thus, considering this structural similarity between PTS proteins and the RPH domains, it can be hypothesized that PTS may represent an evolutionary ancestor of modern rifampicin-modifying enzymes, in a manner analogous to the proposed evolutionary links between penicillin-binding proteins and β-lactamases, or between eukaryotic protein kinases and aminoglycoside acetyltransferases (Hon et al., 1997; Hall and Barlow, 2004).

Interestingly, a recently published study in *B. subtilis* (Sudzinová et al., 2026) identified some of the PTS proteins to be upregulated upon rifampicin treatment, namely (i) the histidine-containing phosphocarrier protein HPr (UniProt P08877) and (ii) the mannitol-specific EIIA permease MtlF (EIIA*mtlF*) (UniProt C0H3V2) (Figure 1C). The involvement of these proteins in rifampicin resistance was previously unknown and was not investigated by Sudzinová et al. The Sudzinova et al. study also revealed that expression of RphT was induced by rifampicin and showed that it contributes to rifampicin resistance. It was speculated (but not shown) that the RphT-mediated resistance could be due to phosphorylation of rifampicin (Sudzinová et al., 2026).

The present work investigates the role of RphT and PTS in rifampicin resistance in *B. subtilis* and establishes a functional parallel between RphT and PTS. RphT is shown to phosphorylate rifampicin with high efficiency, protecting the cell against this antibiotic. Additionally, a PTS component (EI) is demonstrated to possess a rifampicin-phosphorylating activity. This activity relies on ATP, whereas the natural phosphate donor in the PTS cascade is phosphoenolpyruvate (PEP). Three amino acid residues in EI are then identified as important for the phosphorylation activity. However, unlike the rifampicin phosphorylating activity of RphT, the EI-dependent rifampicin phosphorylation does not provide detectable protection for the cell against rifampicin. Rather, it represents a functional remnant, revealing an evolutionary link between seemingly unrelated proteins (sugar transport vs antibiotic inactivation). Finally, overexpressing RphT in *B. subtilis* as well as in a heterologous host enables assessment of the risks of silent RphT homologs becoming “super-resistant” determinants if derepressed or horizontally transferred.

## 2 Materials and methods

### 2.1 Bacterial strains, plasmids, and proteins

All bacterial strains, proteins, and plasmids used in this study are listed in Table 1.

**Table 1:** Bacterial strains, plasmids and proteins used in this study.

Name	Construct	Description	ATB resistance	Reference
*B. subtilis*				
LK4269	wt	*B. subtilis* BSB1 parental wild-type strain	none	(Nicolas et al., 2012)
BKE18830	Δ*rphT*	*B. subtilis*168 *rphT(pps)*: MLS	MLS	(Koo et al., 2017)
BKE13900	Δ*ptsH*	*B. subtilis*168 *ptsH*: MLS	MLS	(Koo et al., 2017)
BKE13910	Δ*ptsI*	*B. subtilis*168 *ptsI*: MLS	MLS	(Koo et al., 2017)
BKE30560	Δ*pckA*	*B. subtilis*168 *pckA*: MLS	MLS	(Koo et al., 2017)
BKE03982	Δ*mtlF*	*B. subtilis*168 *mtlF*: MLS	MLS	(Koo et al., 2017)
LK3162	Δ*rphT*	BSB1 *rphT*: MLS	MLS	This work
LK3800	Δ*ptsH*	BSB1 *ptsH*: MLS	MLS	This work
LK3563	Δ*ptsI*	BSB1 *ptsI*: MLS	MLS	This work
LK3363	Δ*pckA*	BSB1 *pckA*: MLS	MLS	This work
LK3524	Δ*mtlF*	BSB1 *mtlF*: MLS	MLS	This work
LK3968	Δ*rphT amyE:rph* OE	BSB1 *rphT*: MLS *amyE*: P_hyperspank_-*rphT*	MLS, SPC	This work
LK4902	Δ*ptsH amyE:ptsH*	BSB1 *ptsH*: MLS *amyE*: P*_ptsH_*-*ptsH*	MLS, SPC	This work
LK4993	Δ*ptsH amyE:ptsHI*	BSB1 *ptsH*: MLS *amyE*: P*_ptsH_*-*ptsHI*	MLS, SPC	This work
LK4214	Δ*ptsH amyE:ptsGHI*	BSB1 *ptsH*: MLS *amyE*: P*_ptsG_*-*ptsGHI*	MLS, SPC	This work
LK3761	∆*ptsI amyE:ptsI*	BSB1 ptsI: MLS amyE: P_hyperspank_-*ptsI*	MLS, SPC	This work
LK4990	∆*ptsI amyE:ptsHI*	BSB1 *ptsI*: MLS *amyE*: P*_ptsH_*-*ptsHI*	MLS, SPC	This work
LK4911	∆*ptsI amyE:ptsGHI*	BSB1 *ptsI*: MLS *amyE*: P*_ptsG_*-*ptsGHI*	MLS, SPC	This work
LK4387	wt RIF^R^	BSB1 *rpoB*: C1444T (rifampicin resistant)	MLS	This work
LK3743	∆*rphT* RIF^R^	BSB1 ∆*rphT*: MLS *rpoB*: C1444T (rifampicin resistant)	MLS	This work
*E. coli*				
LK625	BL21 (DE3)	*E. coli* BL21 (DE3) wild type	Amp	Laboratory strain
LK3855	RphT-C-His	BL21(DE3) pET22b(P_T7_-*rphT*-6xHis)	Amp	This work
LK4318	RphT H825A-C-His	BL21(DE3) pET22b(P_T7_-*rphT* H825A-6xHis)	Amp	This work
LK3859	HPr-C-His	BL21(DE3) pET22b(P_T7_-*ptsH*-6xHis)	Amp	This work
LK3917	EI-C-His	BL21(DE3) pET22b(P_T7_-*ptsI*-6xHis)	Amp	This work
LK3996	PckA-C-His	BL21(DE3) pET22b(P_T7_-*pckA*-6xHis)	Amp	This work
LK3759	MtlF-C-His	BL21(DE3) pET22b(P_T7_-*mtlF*-6xHis)	Amp	This work
LK4579	EI-H189A-C-His	BL21(DE3) pET22b(P_T7_-*ptsI* H189A-6xHis)	Amp	This work
LK4587	EI-S34A-C-His	BL21(DE3) pET22b(P_T7_-*ptsI* S34A-6xHis)	Amp	This work
LK4578	EI-S36A-C-His	BL21(DE3) pET22b(P_T7_-*ptsI* S36A-6xHis)	Amp	This work
Plasmids				
pLK459	pDR110	plasmid for integration of constructs of interest under P*_hyperspank_* (IPTG inducible) and Spc resistance gene into *amyE* site into *B. subtilis* genome	Amp	(Meijer and Miguel-Arribas, 2024)
pLK3897	*ptsH* in *amyE* OE	pDR110 with P*_hyperspank_*-*ptsH*	Amp	This work
pLK3896	*rphT* in *amyE* OE	pDR110 with P*_hyperspank_*-*rphT*	Amp	This work
pLK4015	*ptsI* in *amyE* OE	pDR110 with P*_hyperspank_*-*ptsI*	Amp	This work
pLK4244	*ptsH* in *amyE* com	pDR110 with P*_ptsH_*-*ptsH*	Amp	This work
pLK4996	*ptsHI* in *amyE* com	pDR110 with P*_ptsH_*-*ptsHI*	Amp	This work
pLK4241	*ptsGHI* in *amyE* com	pDR110 with P*_ptsG_*-*ptsGHI*	Amp	This work

Name	Other names used in literature or in databases	Gene	UniProt number	Description
Proteins—*B. subtilis*				
RphT	Pps, Rph	*rphT, pps*	O34309	Rifampicin phosphotransferase
HPr	PtsH	*ptsH*	P08877	Histidine-containing phosphocarrier protein of the PTS
EI	PtsI	*ptsI*	P08838	Enzyme I, general (non sugar-specific) component of the PTS
PckA	PckA	*pckA*	E0TY79	Phosphoenolpyruvate carboxykinase
MtlF	PtmA, EIIA^mtlF^	*mtlF*	C0H3V2	Mannitol specific permease, EIIA of the PTS

MLS, Macrolide-Lincosamide-Streptogramin B; Spc, spectinomycin; Amp, ampicilin.

### 2.2 Construction of knock-out strains

Genomic DNA of knockout strains from the BKE library (BKE13900, BKE13910, BKE18830, BKE03982, BKE30560 (Koo et al., 2017) was transformed into competent wt BsB1 (Nicolas et al., 2012) cells (LK4269; selection for MLS), resulting in Δ*ptsH* (LK3800), ΔptsI (LK3563), Δ*rphT* (LK3162), Δ*mtlF* (LK3524), Δ*pckA* (LK3363). The resulting strains were confirmed by PCR and sequencing of the deleted gene region.

### 2.3 Construction of complementation/overexpression strains in *B. subtilis*

The *rphT*, *ptsH*, and *ptsI* coding regions including their native stop codon, were PCR-amplified from wt *B. subtilis* genomic DNA (LK4269). The fragments were cloned into pDR110 (pLK459) vector to generate plasmids expressing IPTG-inducible RphT or EI proteins (pLK3896, pLK3897, and pLK4015), which were integrated into the *amyE* locus of the *B. subtilis* chromosome, resulting in strains LK3968 and LK3761.

Complementation of ∆*ptsH* by *ptsH*, *ptsH*-*ptsI* or *ptsG*-*ptsH*-*ptsI* operons under their native promoters was performed by cloning the complete gene or operon regions, including the promoter into pDR110 while omitting the Phyperspank promoter, yielding plasmids pLK4244, pLK4996, and pLK4241. These plasmids were integrated into the *amyE* locus of the ∆*ptsH* strain and selected for spectinomycin to generate the complementation strains under native gene regulation (LK4902, LK4993, LK4214, see Table 1). Plasmids pLK4996 and pLK4241 were also used for complementation of ∆*ptsI* by *ptsH*-*ptsI* or *ptsG*-*ptsH*-*ptsI* operons under their native promoters. These plasmids were integrated into the *amyE* locus of the ∆*ptsI* strain, and spectinomycin-resistant colonies were selected, yielding strains LK4990 and LK4911, see Table 1. All the plasmids and strains were verified by sequencing.

### 2.4 Rifampicin-resistant *B. subtilis*/Δ*rphT*

Wild type (LK4269) or Δ*rphT* (LK3162) cells were streaked on LB agar and incubated overnight at 37 °C. A single colony was inoculated into 10 mL of LB and grown overnight at 37 °C, then subcultured to an OD600 0.1 in 10 mL of LB and grown to an OD600 ~ 1.0. An aliquot (200 μL) was plated on an LB agar supplemented with 1 μg/mL rifampicin. After overnight incubation, several colonies were selected for gDNA isolation and *rpoB* sequencing. A strain (LK4387) carrying the C1444T mutation in *rpoB*, causing the H482Y substitution in the β subunit, was selected for further work. The same mutation was isolated in the Δ*rphT* background (LK3743). This mutation confers rifampicin resistance (Leehan and Nicholson, 2021).

### 2.5 Media, growth conditions, and antibiotics

Cells were grown in LB medium (Miller) at 37 °C with vigorous shaking. For growth on solid media, LB plates containing 1.5% agar were used. Antibiotics were added at the following concentrations: ampicillin 100 μg/mL, MLS (erythromycin 0.5 μg/mL plus lincomycin 12.5 μg/mL), and spectinomycin 100 μg/mL. Rifampicin stocks were freshly diluted in ethanol prior to each experiment and adjusted with sterile water to 30 μg/mL. Then the diluted rifampicin was added to the LB medium to a final concentration of 0.03 μg/mL. This concentration was determined experimentally as ½ MIC for rifampicin in *B. subtilis* and was referred to as a subinhibitory concentration of this antibiotic.

### 2.6 Phenotypic experiments in liquid or on solid media

Strains were grown overnight on LB agar plates supplemented with appropriate antibiotics. A single colony was used to inoculate overnight LB cultures, which were subsequently diluted into fresh medium and grown for 6 h before initiating experiments. For liquid growth assays, LB or LB with 0.03 μg/mL rifampicin was inoculated to an OD600 = 0.03 in 96-well plates and monitored for 15 h at 37 °C with shaking in a Spark Multimode Microplate reader (Tecan). For assays on solid medium, the cells were adjusted to OD600 = 1, serially diluted into LB without antibiotics (down to 10−5) and spotted onto LB agar complemented with IPTG or LB agar complemented with IPTG and 0.03 μg/mL rifampicin using a Replica plater for 96 well plate (Sigma-Aldrich). Plates were incubated overnight at 37 °C. All experiments were performed in at least three independent replicates. The doubling times were calculated using the software available at: https://dashing-growth-curves.ethz.ch/. For statistical comparison one-way Anova test was used (GraphPad Prism).

### 2.7 Protein purification

Plasmids overexpressing recombinant proteins in *E. coli* were constructed by inserting *rphT*, *ptsI*, *ptsH*, *mtlF*, or *pckA* gene into pET-22b using Gibson assembly (New England Biolabs). The resulting vectors positioned each coding sequence 8 bp downstream of the ribosome binding site and are fused directly to a C-terminal 6x His-tag. Single-point mutants (coding RphT H825A, EI H189A, EI S34A, and EI S36A) were generated by PCR-based mutagenesis. All constructs were sequence-verified and transformed into *E. coli* BL21 (DE3) yielding strains LK3855 (RphT-C-His6), LK3996 (PckA-C-His6), LK3759 (MtlF-C-His6), LK3859 (HPr-C-His6), LK3917 (EI-C-His6), LK4318 (RphT H825A-C-His6), LK4579 (EI-H189A-C-His), LK4587 (EI-S34A-C-His) and LK4578 (EI-S36A-C-His).

Cultures were grown in 1 L LB at 37 °C to OD600 = 0.5, induced with 0.75 mM IPTG, and incubated for 2 h at room temperature with shaking. Cells were harvested by centrifugation (10 min, 6,500 g, 4 °C), the pellet was washed with 1x Buffer (50 mM Tris–HCl, pH 8; 300 mM NaCl). After centrifugation (7,690 g, 10 min, 4 °C) pellet was resuspended in lysis buffer (50 mM Tris–HCl, pH 8; 300 mM NaCl; 10 mM imidazole, 5 mM β-mercaptoethanol, protease inhibitor cocktail (Thermo Scientific) and lysed by sonication (Bandelin sonopuls, VS 70 T probe, 15 × 10 s on ice with 1-min pauses between cycles, amplitude 50%). Lysates were clarified by centrifugation (7,690 g, 10 min, 4 °C) and filtered through a 0.22 μm filter (Millex-GV, Merck). Filtrated lysate was applied to a 1 mL His-Trap HP column (Cytiva) and washed with 5 mL washing buffer (20 mM Tris–HCl, pH = 8; 300 mM NaCl; 10% glycerol; 20 mM imidazole). Bound proteins were eluted with elution buffer (50 mM Tris–HCl, pH 8; 150 mM NaCl; 400 mM imidazole), pooled and dialyzed into storage buffer (50 mM Tris–HCl pH 8.0; 150 mM NaCl, 5 mM β-mercaptoethanol, 50% glycerol), and stored at –20 °C until further use. Protein purity was assessed by SDS-PAGE (NuPAGE 4%–12% Bis-Tris Gel, Invitrogen).

### 2.8 Rifampicin phosphorylation assay

The same amount of purified *B. subtilis* PTS proteins (25 μM each) or purified RphT (2.5 μM, positive control) was mixed with rifampicin (10 μg/mL) and ATP (1 mM) or PEP (10 μM) in the presence of reaction buffer (50 mM Tris–HCl, pH 7.6; 5 mM MgCl2; 40 mM NH4Cl). The mixture was incubated for 1 h at 37 °C and protected from light. *In vitro* reaction mixtures were then subjected to protein precipitation using cold (−20 °C) acetonitrile (ACN): twice the sample volume of ACN (2:1 ACN:sample) was added, and the mixtures were vortexed and centrifuged for 10 min (20,627 g, 4 °C). The protein pellet was discarded, the supernatant was filtered through 3 kDa Amicon Ultra cartridges (Millipore, United States) (30 min, 20,627 x g, 4 °C), and the flow-through was analyzed using LC–MS.

### 2.9 LC–MS analysis

Liquid chromatography mass spectrometry (LC–MS) analyses were performed on an Agilent 1,290 LC system (Agilent, USA) coupled to a TimsTOF HT mass spectrometer (Bruker, Germany). Separation was achieved on a Waters CSH C18 Premier LC column (100 mm × 2.1 mm, 1.7 μm) at 40 °C. Three microliters of sample were injected and eluted at 0.4 mL/min using a two-component mobile phase consisting of 0.1% formic acid in water (A), and ACN (B), with a linear gradient: (min/%B) 0/5; 1.5/5; 10/46; 15/100, followed by 1 min column wash (100% B) and 3 min re-equilibration (5% B). The mass spectrometer operated in positive ionization mode with a capillary voltage of 4.5 kV, desolvation gas temperature of 220 °C, end plate offset of 500 V; desolvation gas flow of 10 L/min, scan rate of 12 Hz, and total cycle time of 0.50 s. A mixture of Agilent tune mix (Agilent, USA) and sodium formate (3:1, v/v) was used for calibration at the beginning of each injection. Mass spectra were collected in the 20–1,300 m/z range with typical mass accuracy below 0.005 Da. The data was processed with DataAnalysis (Bruker, Germany) by extracting ion chromatograms corresponding to the [M + H]^+^ ions of the analytes with a mass tolerance of 0.005 Da. Each analysis was repeated at least three times in three independent experiments. The primary data were deposited at https://massive.ucsd.edu/ProteoSAFe/dataset.jsp?accession=MSV000100512.

### 2.10 Culture broth supernatants and cell lysates

Appropriate strains (LK4269, LK3162, LK4387, LK3743, see Table 1) were grown in liquid LB media until mid exponenetial phase (OD600 = 0.5). Then rifampicin (final concentration 0.03 μg/mL for wt and Δ*rphT* strains or 10 μg/mL for rifampicin-resistant strains) was added, and the cultures were incubated with shaking at 37 °C for one additional hour. Then the culture was centrifuged (7,690 x g, 10 min, 4 °C, Hettich 320R centrifuge). Culture supernatant was used for LC–MS analysis as described below.

Pelet was washed with 10 mL of wash buffer (50 mM Tris–HCl, pH 7.5, 50 mM NaCl) and after centrifugation (7,690 x g, 10 min, 4 °C, Hettich 320R centrifuge), the pelet was resuspended again in 10 mL of wash buffer containing 1 mg/mL lysozyme (Serva). The mixtures were incubated for 10 min at 37 °C and then sonicated (Bandelin sonopuls, VS 70 T probe, 10×10 s on ice with 1 min pause between cycles, 50% amplitude). After centrifugation, the supernatant was kept as “cell lysate.”

Samples (culture supernatant as well as cell lysates) were extracted using solid-phase extraction with Oasis HLB 3 cc, 60 mg cartridges (Waters, USA). The cartridges were conditioned with 3 mL of methanol followed by 3 mL of Milli-Q water (Millipore, USA). Subsequently, 3 mL of the sample was loaded onto the cartridge, which was then washed with 3 mL of Milli-Q water. Analytes were eluted with 5 mL of methanol, and the eluate was dried at ambient temperature using SpeedVac vacuum concentrator (Eppendorf, Germany). The residues were reconstituted in 150 μL of 50% methanol prior to LC–MS analysis, resulting in extracts that were 20 × preconcentrated compared to the original samples.

### 2.11 HPr phosphorylation assay

HPr (33 μM) was incubated with 0.8 μM wt or phosphoablative EI variants in phosphorylation buffer (50 mM Tris–HCl pH 8, 10 mM MgCl_2_) in the presence of 1 mM ATP or 10 μM PEP (concentration sufficient for full phosphorylation of the same amount of HPr by HprK phosphotransferase, data not shown). Reactions were incubated 30 min at 37 °C, mixed with 4x Native loading dye (Invitrogen) and loaded on NativePAGE 4 to 16% Bis-Tris Gels (Invitrogen). Gels were run at room temperature in 1x Native running buffer for 1 h at 150 V and stained with SimplyBlue SafeStain (Invitrogen).

### 2.12 Minimal inhibitory concentration (MIC) in *E.Coli*

For MIC experiments, strains LK625, LK3855, LK3859, and LK3917 (see Table 1) were used. Strains were grown overnight on LB agar with appropriate antibiotics. A single colony was used to inoculate overnight LB cultures, which were diluted into fresh medium and grown for 6 h before MIC testing. MICs were determined in 96-well plates with 100 μL volume per well. Cells were diluted to a final concentration of 5 × 10⁵ CFU/ml and exposed to two-fold serial dilutions of rifampicin in the presence or absence of 0.5 mM IPTG. The highest tested concentration was 200 μg/mL. Plates were incubated for 24 h at 37 °C. After that, 4 μL of 0.8 mg/mL resazurin (Sigma-Aldrich) was added to each well to distinguish wells containing growing cells from wells with dead cells. Plates were incubated for an additional 2 h before imaging. Experiments were performed in at least three independent replicates.

### 2.13 Minimal inhibitory concentration (MIC) in *B. subtilis*

For MIC experiments in *B. subtilis* strains LK4269, LK3162, and LK3968 (see Table 1) were used. The procedure was the same as for MIC in *E. coli* except that the highest rifampicin concentration tested was 0.96 μg/mL. Experiments were performed in at least three independent replicates.

### 2.14 Nano-DSF

All measurements were done using a Prometheus NT.48 and Prometheus Standard Capillaries (NanoTemper Technologies). The final concentration of EI in each measurement was 0.5 mg/mL. The first measurement was done in 50 mM Bis-Tris pH 6.5 with 50 mM NaCl, temperature range from 22 °C to 80 °C, using a 1.5 °C/min gradient, 100% excitation, and in duplicates. The second measurement was done in 40 mM Tris–HCl, pH 8.0, 50 mM KCl, and 10 mM MgCl_2_, temperature range 22 °C to 85 °C, gradient 1.5 °C/min, and excitation 90%. Data were measured, processed and figures were created using the PR.ThermControl software v2.11 (NanoTemper Technologies).

### 2.15 Microscale thermophoresis

All Microscale Thermophoresis (MST) measurements were performed using a Monolith NT.115 instrument with Monolith Standard Capillaries MO-K022 (NanoTemper Technologies), the red excitation laser set to 30%, and the manual temperature control set to 22 °C. The MST power was set to 50%. Before measuring thermophoresis, fluorescence was monitored for 5 s. Then, the MST excitation (infrared laser) was turned on for 25 s, and the fluorescence after MST was measured for 5 s. The delay between individual measurements was set to 25 s. EI was labeled using the His-Tag Labeling Kit RED-Tris-NTA 2nd Generation (NanoTemper Technologies) in the assay buffer with a 2:1 molar excess of EI and a 30 min reaction time before the measurements.

Measurements were performed three times using the same batch of EI and rifampicin. However, EI was freshly labeled before each series. A 1:1 serial dilution of rifampicin was used. The final concentration of EI in all measurements was 50 nM, with the estimated labeled EI concentration 25 nM. All titration series were mixed independently (30 min reaction time) before measurements. The first two titration series were performed in an assay buffer of 50 mM Hepes (pH 7.5), 50 mM NaCl, and 0.1% (w/v) Pluronic F-127. The third series was performed in the same buffer with the addition of 2.5% (v/v) DMSO. In the first two series, the rifampicin concentration ranged from 500 μM to 61 nM. In the third series, the rifampicin concentration ranged from 2,500 μM to 610 nM. Data was processed using the MO.Affinity Analysis software v2.2.4 (NanoTemper Technologies). The full report generated by the software is included in the Supplementary material. The raw MST data were deposited in the Molecular Biophysics Database (Agerschou et al., 2025) under doi: 10.71479/gf2bm-df854.

## 3 Results

### 3.1 Deletion of genes encoding RphT, HPr, or EI compromises growth under rifampicin stress

To begin evaluating the functional relevance of the PTS proteins in rifampicin resistance, clean in-frame deletions of genes encoding RphT, and PTS proteins sharing structural or functional homology with RPH were generated (see Methods and Table 1). Specifically, the deletions were in genes encoding RphT (*rphT* gene), and PTS proteins HPr (*ptsH* gene), EI (*ptsI* gene), MtlF (*mtlF* gene), and PckA (*pckA* gene). PEP synthase PckA was selected as a functional homolog of the RPH ATP-grasp domain, because no structural homolog is present in *B. subtilis*.

**Figure 2:**
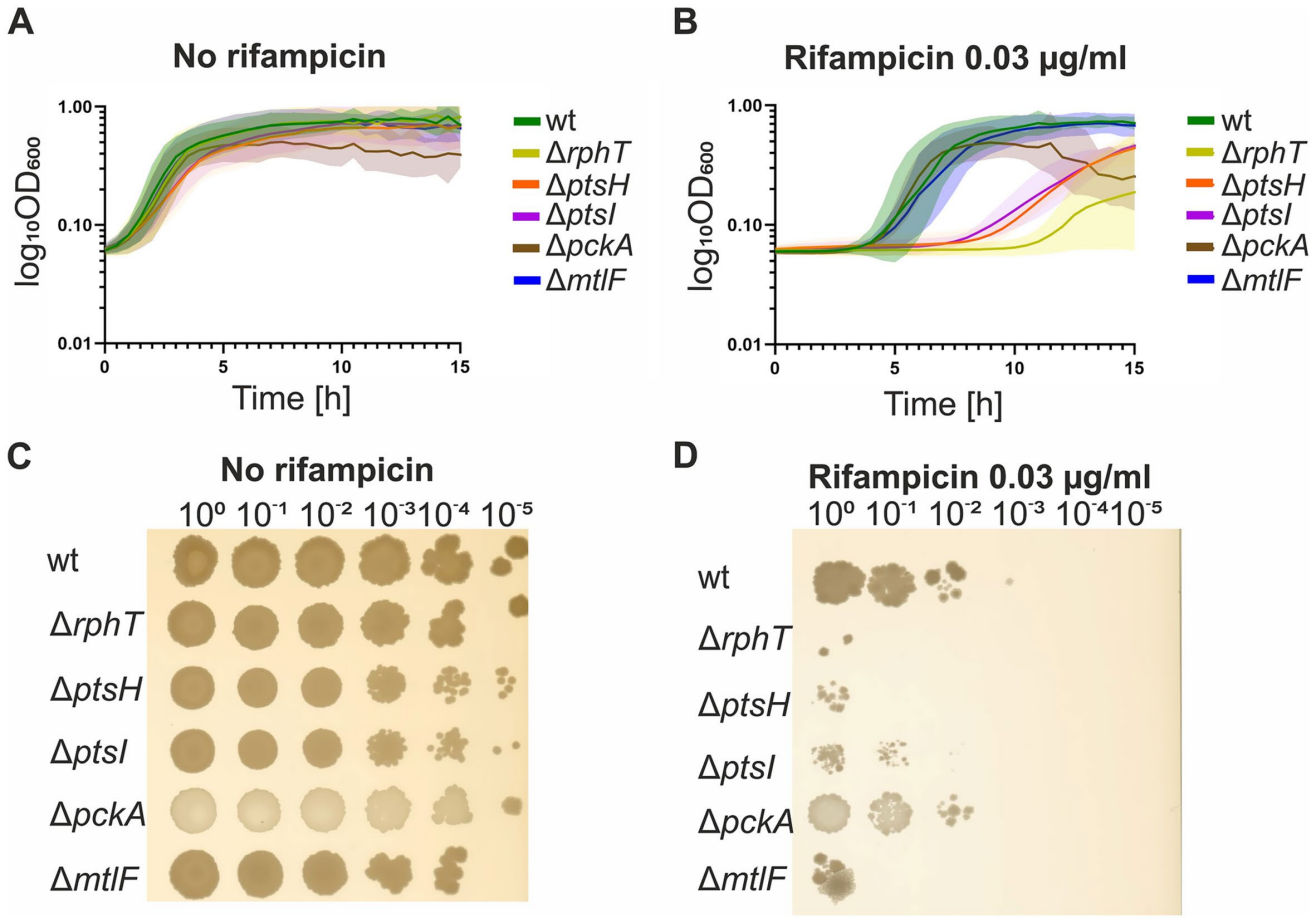
Growth of *B. subtilis* WT and deletion strains in the absence and presence of subinhibitory rifampicin (0.03 μg/mL). Panels **(A)** and **(B)** show growth in liquid LB medium in 96-well plates; averages of three biological repetitions are presented, standard deviations from three independent experiments are depicted as shadow areas. Panels **(C)** and **(D)** show growth on solid medium. A representative plate from three independent experiments for each of the conditions is shown. Dilutions of the starting culture of OD_600_ = 0.6 (corresponding to 10^0^) are indicated above the figure. *rphT*, gene encoding enzyme rifampicin phosphotransferase; *ptsH*, gene encoding HPr protein; *ptsI*, gene encoding EI protein; *pckA*, gene encoding PckA protein; *mtlF*, gene encoding MtlF protein.

In liquid medium, mutants displayed wild-type (wt) growth characteristics in LB without rifampicin until the early stationary phase, only Δ*ptsH* exhibited slightly longer doubling times (Figure 2A, Supplementary Table S1). In the presence of a subinhibitory rifampicin concentration (0.03 μg/mL) the Δ*rphT* strain exhibited the most severe defect (dose-dependent growth in liquid media, manifested by prolonged lag-phase, and slow growth phenotype on solid media (Dabbs et al., 1995). Δ*ptsH* and Δ*ptsI* exhibited intermediate yet reproducible growth defects (longer lag phase and longer doubling time), whereas Δ*mtlF* and Δ*pckA* displayed growth kinetics comparable to the wild type until the onset of late stationary phase (Figure 2B, Supplementary Table S1). During late stationary phase, the Δ*pckA* strain showed an accelerated decline in viability. However, this phenotype did not affect the dose-dependent lag phase length, which reflects the rifampicin adaptability.

Spot-dilution assays on solid medium (Figures 2C,D) showed that, in the presence of rifampicin, Δ*rphT* displayed the strongest inhibition; Δ*ptsH* and Δ*ptsI* grew less than wt, while Δ*pckA* behaved comparably to the wt (Figure 2D). The Δ*mtlF* strain, however, displayed a slow growth phenotype on solid media compared to wt.

Taken together, these phenotypic observations indicate that apart from RphT, PTS proteins EI, HPr, and (at least on solid media) MtlF are required for optimal tolerance to this antibiotic.

### 3.2 Genetic complementation restores rifampicin tolerance

To validate that the growth defects of Δ*rphT*, Δ*ptsH* and Δ*ptsI* were due to the respective gene deletions, the corresponding wt alleles were reintroduced at the neutral *amyE* locus to perform complementation experiments. The Δ*rphT* strain was complemented with an IPTG-inducible copy of *rphT*. As *ptsH* and *ptsI* can be transcribed as two different transcriptional units (*ptsHI* and *ptsGHI*) from two different promoters and possess an overlapping sequence important for translational coupling, three complementation constructs were built under their native promoters: (i) *ptsH*, (ii) the *ptsH*-*ptsI* transcriptional unit, and (iii) the full *ptsG*-*ptsH*-*ptsI* transcriptional unit (Supplementary Figure S1). Single gene complementation of *ptsI* was tested only with an IPTG-inducible promoter.

**Figure 3:**
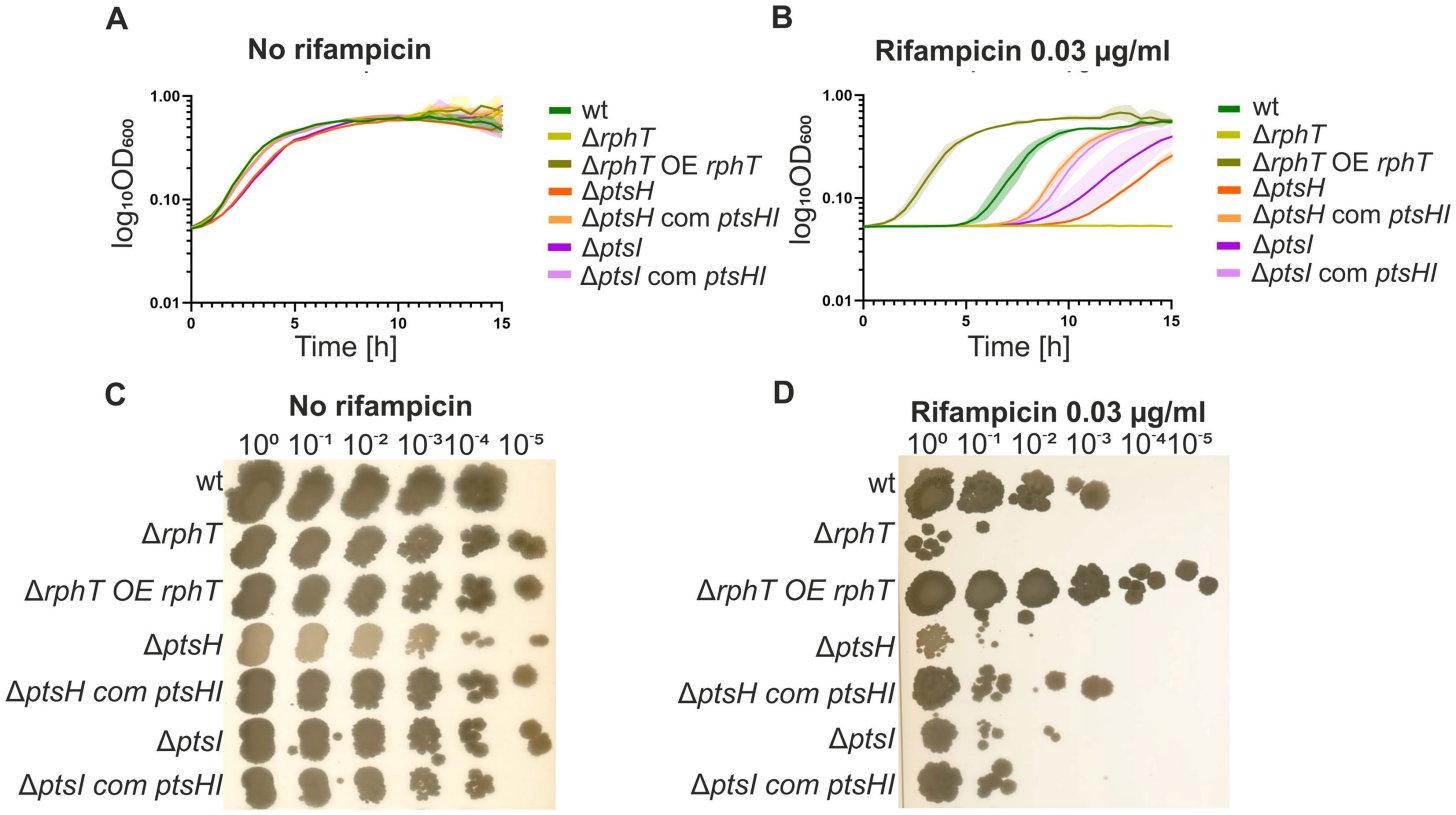
Growth of *B. subtilis wt*, *rphT, ptsH,* and *ptsI* deletion strains and the respective complementation strains in the absence and presence of subinhibitory concentrations of rifampicin (0.03 μg/mL). **(A, B)** The growth was performed in liquid LB medium in 96-well plates as described in Materials and Methods, averages of two biological repetitions are presented, range of the values are depicted as shadow areas. For better clarity only complementation’s by the ectopic *ptsHI* are shown. Panels **(C)** and **(D)** show growth on solid medium. A representative plate from three independent experiments for each of the conditions is shown. Dilutions of starting culture of OD_600_ 0.6 are indicated above the figure. OE, overexpression; com, complementation.

In liquid cultures containing rifampicin, the Δ*rphT*-complemented strain completely rescued the wt phenotype and even surpassed it at high IPTG concentrations (Figures 3A,B). Complementation of Δ*ptsH* with *ptsH* or Δ*ptsI* by inducible *ptsI* did not restore the wt phenotype (data not shown), probably due to the complex expression regulation of the two genes (deletion of one likely affected expression of the other). Complementation of Δ*ptsH* or Δ*ptsI* with the *ptsHI* operon (or the larger *ptsGHI* unit) partially improved growth relative to the deletion mutants (Figures 3A,B; Supplementary Figure S2). Spot assays yielded similar results (Figures 3C,D), confirming that HPr and EI contribute to rifampicin tolerance.

### 3.3 PTS proteins phosphorylate rifampicin *in vitro*

**Figure 4:**
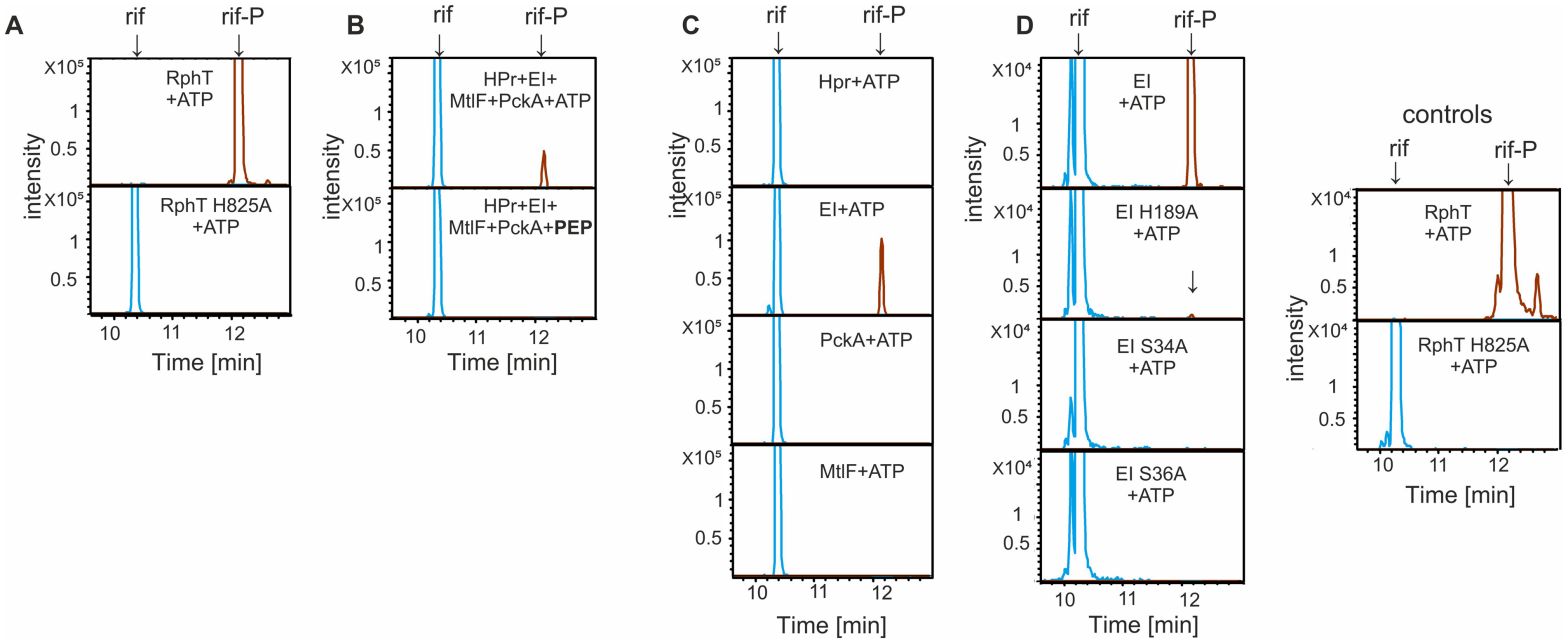
LC–MS chromatograms of *in vitro* phosphorylation of rifampicin (10 μg/reaction) by PTS proteins and RphT in the presence of ATP or PEP. Rifampicin was present in all reactions; the presence of the proteins and cofactors is indicated in each panel. Peaks corresponding to rifampicin (rif) and phosphorylated rifampicin (rif-P) are marked by arrows. **(A)** RphT and RphT H825A (phosphoablative form). These reactions served as positive/negative controls for the following *in vitro* rifampicin assays. **(B)** Reactions with complex of four PTS proteins (HPr, EI, MtlF, PckA) and either ATP or PEP (PEP is bold to distinguish it from other reactions where ATP was used as phosphate donor). **(C)** Reactions with individual PTS proteins and ATP as phosphate donor. **(D)** EI wild-type and its three phosphoablative variants (EI H189A, S34A, S36A). All LC–MS analyses in **(A–D)** were performed in triplicate across three independent experimental runs. Representative image is shown. Extracted ion chromatograms for RIF (*m/z* 823.41) and RIF-P (*m/z* 903.37) [M + H]^+^ are displayed.

To start examining whether the PTS complex could directly modify rifampicin, we established a positive control, *B. subtilis* RphT. This protein shares 62% amino acid identity with RPH from *Listeria monocytogenes* (Stogios et al., 2016). We expected that the *B. subtilis* RphT homologue would also possess rifampicin phosphorylating activity dependent on ATP. Formation of the product (rif-P) was monitored with liquid chromatography-mass spectrometry (LC–MS), with results presented as ion extracted chromatograms (Figure 4) and supported with MS and MS/MS spectra (Supplementary Figures 3A,B). Indeed, *B. subtilis* RphT readily converted rifampicin to the phosphorylated product while a catalytic site mutant of RphT (H825A) showed no conversion (Figure 4A).

Next, purified *B. subtilis* proteins HPr, EI, MtlF, and PckA were combined and incubated with rifampicin, and either ATP (the phosphate donor used by RphT) or phosphoenol-pyruvate (PEP, the physiological donor for the PTS). We detected rif-P in the reactions only when ATP was used as a phosphate donor, but not with PEP (Figure 4B). The results revealed that the PTS protein complex possesses the ability to phosphorylate rifampicin, although the level of rif-P was lower than in the case of RphT.

### 3.4 Enzyme EI is responsible for rifampicin phosphorylation *in vitro*

To investigate which component of the PTS proteins (HPr, EI, MtlF and PckA) is required for rifampicin phosphorylation, we performed *in vitro* reactions and LC–MS analyses with individual PTS proteins in the presence of ATP. From the four PTS proteins, only EI generated a detectable rif-P peak (Figure 4C). HPr, MtlF, and PckA gave no detectable rifampicin modification under the tested conditions. This finding is surprising, as no prior direct connection between EI and antibiotics has been reported so far. Likewise, no prior evidence was reported regarding ATP as a possible phosphate donor for EI phosphorylation.

### 3.5 EI activity verification

Subsequently, we examined which amino acids are important for the observed activity of EI. We created three phosphoablative site-directed mutants of known or predicted phosphorylation sites of EI: (i) H189A (the phospho-histidine required for HPr phosphorylation), (ii) S34A, and (iii) S36A (putative auxiliary phosphorylation sites with unknown functions, situated in the N-terminal EI domain) (Macek et al., 2008).

**Figure 5:**
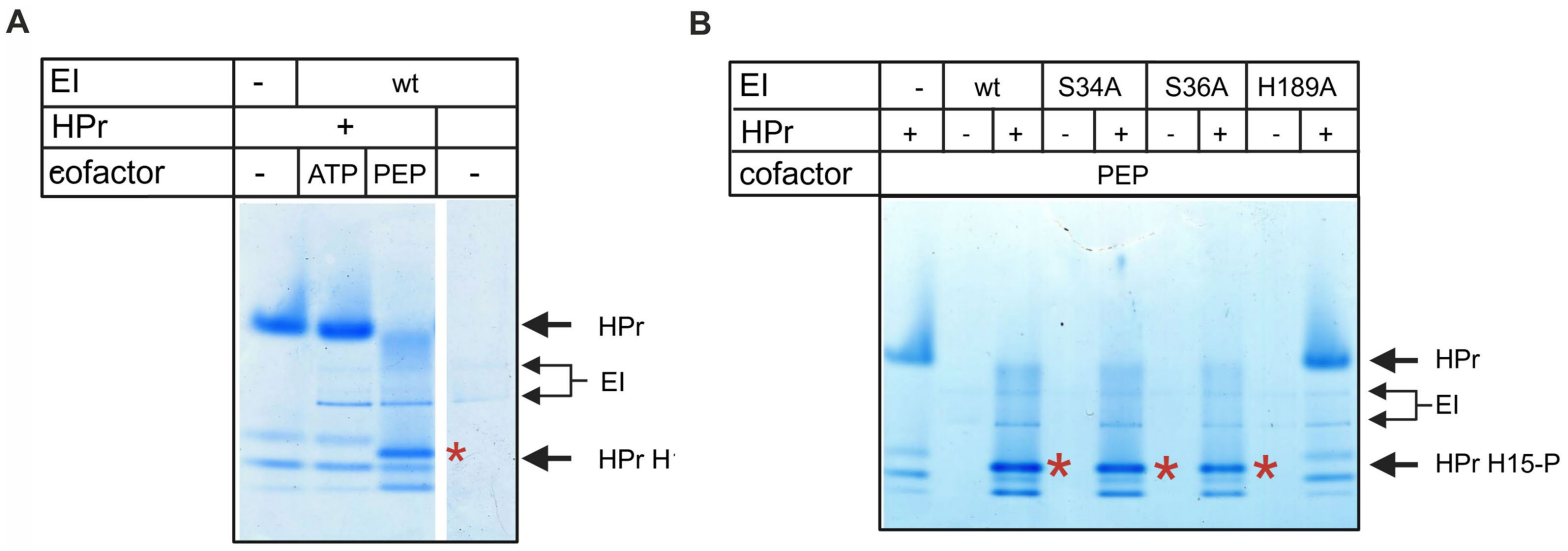
Phosphorylation of HPr by EI. **(A)** Phosphorylation of HPr to HPr-H15P by EI was tested *in vitro* and visualized by native PAGE. Phosphorylation assays contained EI, HPr, and PEP or ATP as indicated in the figure. **(B)** Phosphorylation of HPr to HPr-H15P by EI mutants. Native PAGE gels of *in vitro* phosphorylation assays containing HPr and EI wild type or its phosphoablative forms with PEP in combinations as indicated in the figure. Red asterisks indicate the band corresponding to the H15 phosphorylated form of HPr.

First, we assessed the activity of the EI protein and its mutants in its physiological reaction, i.e., phosphorylation of HPr by EI, in the presence of PEP (Figure 5A). In agreement with the literature (Weigel et al., 1982; Alpert et al., 1985) native-PAGE analyses confirmed that EI phosphorylated HPr in the presence of PEP but not with ATP as the phosphate donor (Figure 5A). Moreover, we verified that EI-H189A failed to phosphorylate HPr in the presence of PEP, whereas the S34A and S36A mutants behaved as wt (Figure 5B). Thus, these experiments confirmed that the EI protein and its mutant versions were functional in physiological reactions depending on the H189 residue and its known cellular partner HPr. In contrast, the S34 and S36 residues were not required for HPr phosphorylation, consistent with no known role of these residues in the PTS phosphotransferase cascade.

All three mutants of EI protein (H189A, S34A, and S36A) together with wt EI were then tested in the *in vitro* rifampicin phosphorylation assay. The phosphoablative mutants S34A and S36A were catalytically inactive, whereas the H189A mutant retained a faint but reproducible residual ability to phosphorylate rifampicin (Figure 4D). If formation of a His189–P covalent intermediate was required for rifampicin modification, substitution of this residue would be expected to completely abolish rif-P formation. Therefore, the results indicate that histidine 189 is not the principal site for rifampicin modification. Furthermore, S34 and S36, possibly undergoing phosphorylation, play an important role in this process.

### 3.6 EI-mediated rifampicin phosphorylation is not detectable *in vivo*

**Figure 6:**
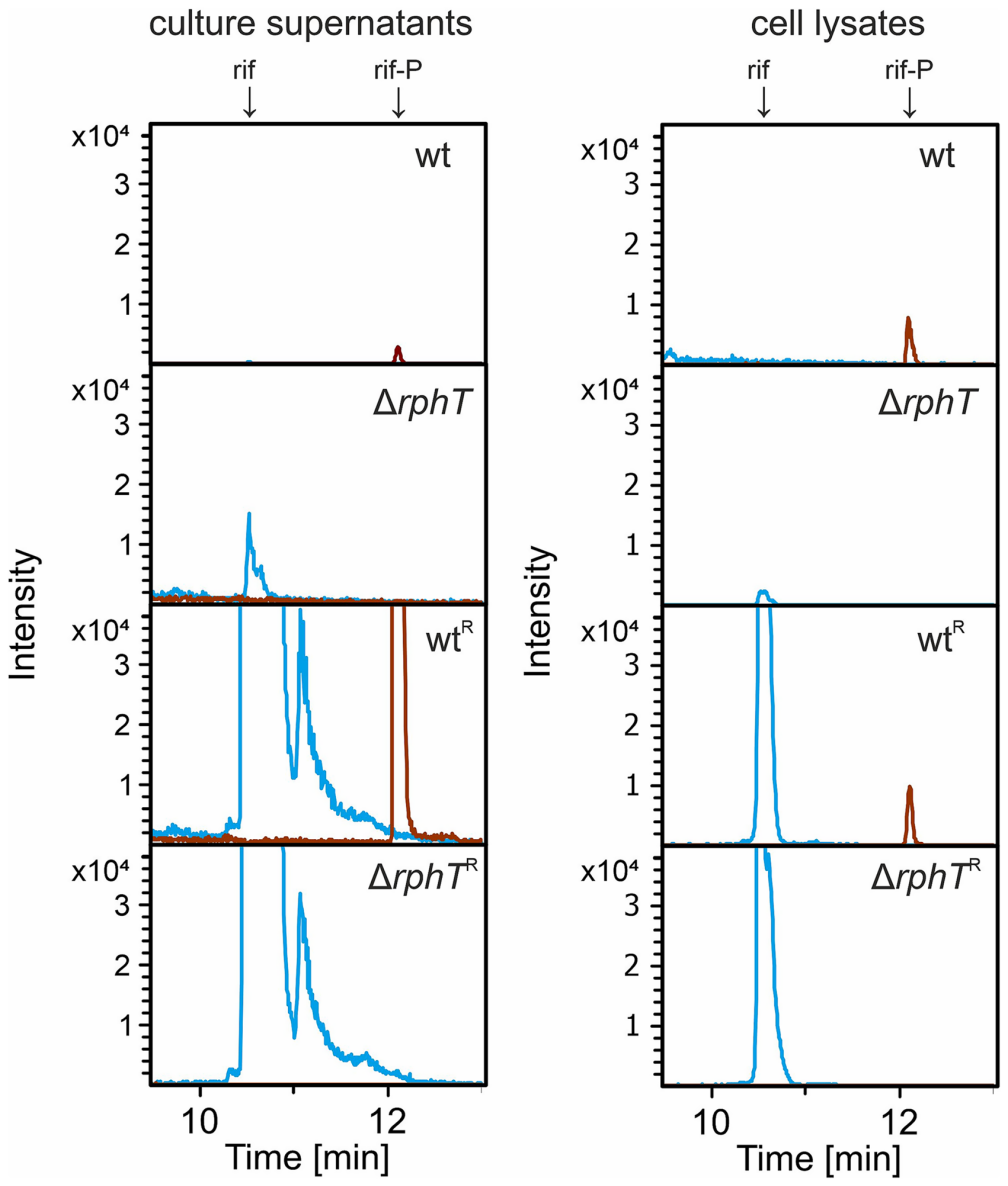
LC–MS analyses of culture supernatants and cell lysates. Samples of *B. subtilis WT, ∆rphT,* and their rifampicin-resistant derivatives (wt^R^—WT strain containing rifampicin resistance mutation, Δ*rphT^R^—rphT* deletion strain containing rifampicin resistance mutation) were analyzed for the presence of rifampicin-phosphate (rif-P). Peaks corresponding to rifampicin and phosphorylated rifampicin are marked by arrows (rif refers to the peak of rifampicin, rif-P refers to the peak of phosphorylated rifampicin). Extracted ion chromatograms for rif (*m/z* 823.41) and rif-P (*m/z* 903.37) [M + H]^+^ are displayed.

To assess whether the *in vitro* observed EI-dependent phosphorylation of rifampicin can be detected *in vivo*, we used LC–MS to analyse rifampicin and rif-P in the culture supernatant and cell-lysate fractions of wt and Δ*rphT* cells grown in LB with a subinhibitory concentration of rifampicin. The expectation was that in wt, RphT is responsible for the majority of rif-P, while in Δ*rphT*, any rif-P would be generated by EI or other yet uncharacterized enzymes. The analyses showed that in wt, rifampicin in both supernatants and lysates had been completely converted to rif-P (Figure 6); on the other hand, no rif-P was detected in Δ*rphT* cells. The same pattern was observed also with rifampicin-resistant strains (harboring *rpoB* mutations) that were employed to culture the cells at high rifampicin concentration (10 μg/mL) to facilitate detection of rif-P: only the wt strain produced detectable rif-P (Figure 6). This indicated that the EI rifampicin phosphorylating activity observed *in vitro* was not detectable in the *in vivo* conditions and is not physiologically relevant.

Interestingly, most of the phosphorylated rifampicin was detected in the culture supernatant indicating the presence of an undefined effective export mechanism. This effect is even more pronounced in the rifampicin-resistant strain (Figure 6), showing that rifampicin undergoes phosphorylation and export despite its inability to bind RNA polymerase and block RNA synthesis.

### 3.7 Direct binding of rifampicin to EI

**Figure 7:**
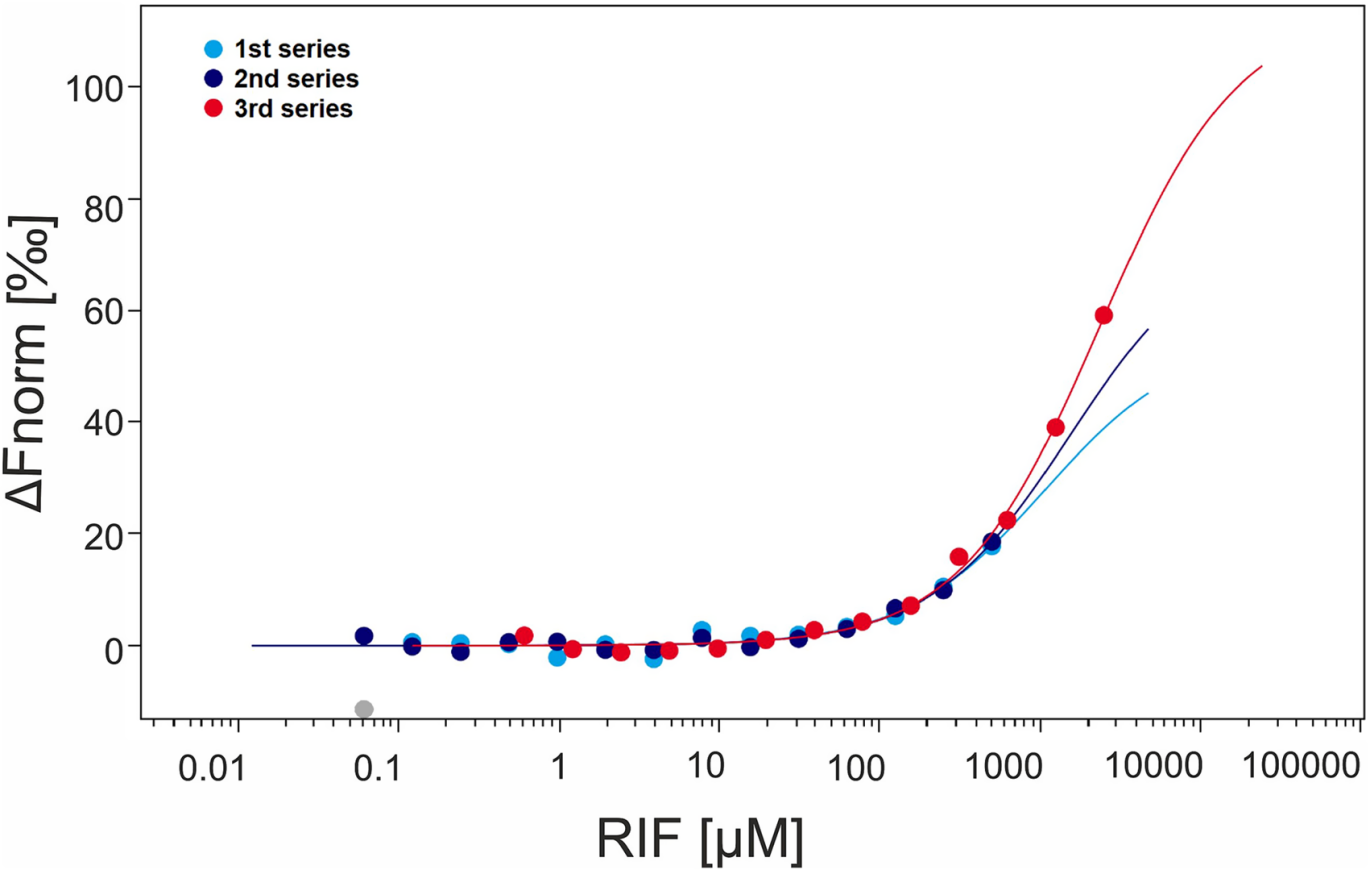
Interaction of EI and rifampicin measured by microscale thermophoresis (MST). The plot displays the normalized fluorescence change (ΔFnorm, ‰) on the *y*-axis as a function of rifampicin concentration (cRIF, μM) on a logarithmic *x*-axis. Each point represents a measurement from one of three independent MST series: two performed with RIF ranging from 61 nM to 500 μM and one with RIF ranging from 610 nM to 2,500 μM. The curves correspond to fits generated by the MO. affinity analysis software v2.2.4. Data for all three series are overlaid to visualize consistency across experiments. The full analysis report is provided in the Supplementary material (Data sheet 1). The figure was generated in the same software and manually edited.

As the *in vivo* experiments failed to detect rifampicin phosphorylation when RphT was absent, we investigated the interaction between rifampicin and EI by Microscale Thermophoresis (MST; Figure 7). Three independent series of measurements confirmed the interaction between EI and rifampicin. However, the affinity was too low (in the millimolar range) to allow reliable determination of the dissociation constant (*K*_d_) under the tested conditions. This weak binding is consistent with the *in vitro* experiments in which the amount of phosphorylated rifampicin generated by EI was two orders of magnitude lower than that produced by the dedicated phosphotransferase RphT. The weak interaction also likely explains why phosphorylated rifampicin was undetectable in the Δ*rphT* strain *in vivo*.

A weak interaction was also observed using nano-Differential Scanning Fluorimetry (nano-DSF). In the presence of rifampicin, the melting temperature (*T*_m_) of EI increased by ∼2 °C (Supplementary Figure S4). The modest thermal stabilization was indicative of a ligand-protein interaction and supported the biochemical data showing that EI can bind rifampicin.

### 3.8 Overexpression of RphT confers high-level rifampicin resistance in a heterologous host

Finally, we were intrigued by the fact that the presence of a highly effective rifampicin deactivation enzyme (i.e., RphT) encoded in the *B. subtilis* genome, as well as in other rifampicin-sensitive bacteria such as *Listeria* and some Actinobacteria (Spanogiannopoulos et al., 2014), stood in contrast to their apparent relative susceptibility to rifampicin. Our recent work showed that RphT expression in *B. subtilis* is inducible by rifampicin and depends on two convergent promoters that enable the cell to sense subinhibitory concentrations of this antibiotic (Sudzinová et al., 2026). This induction is at least an order of magnitude higher compared to conditions without rifampicin, yet still insufficient to confer protection against clinically relevant doses of the antibiotic.

**Figure 8:**
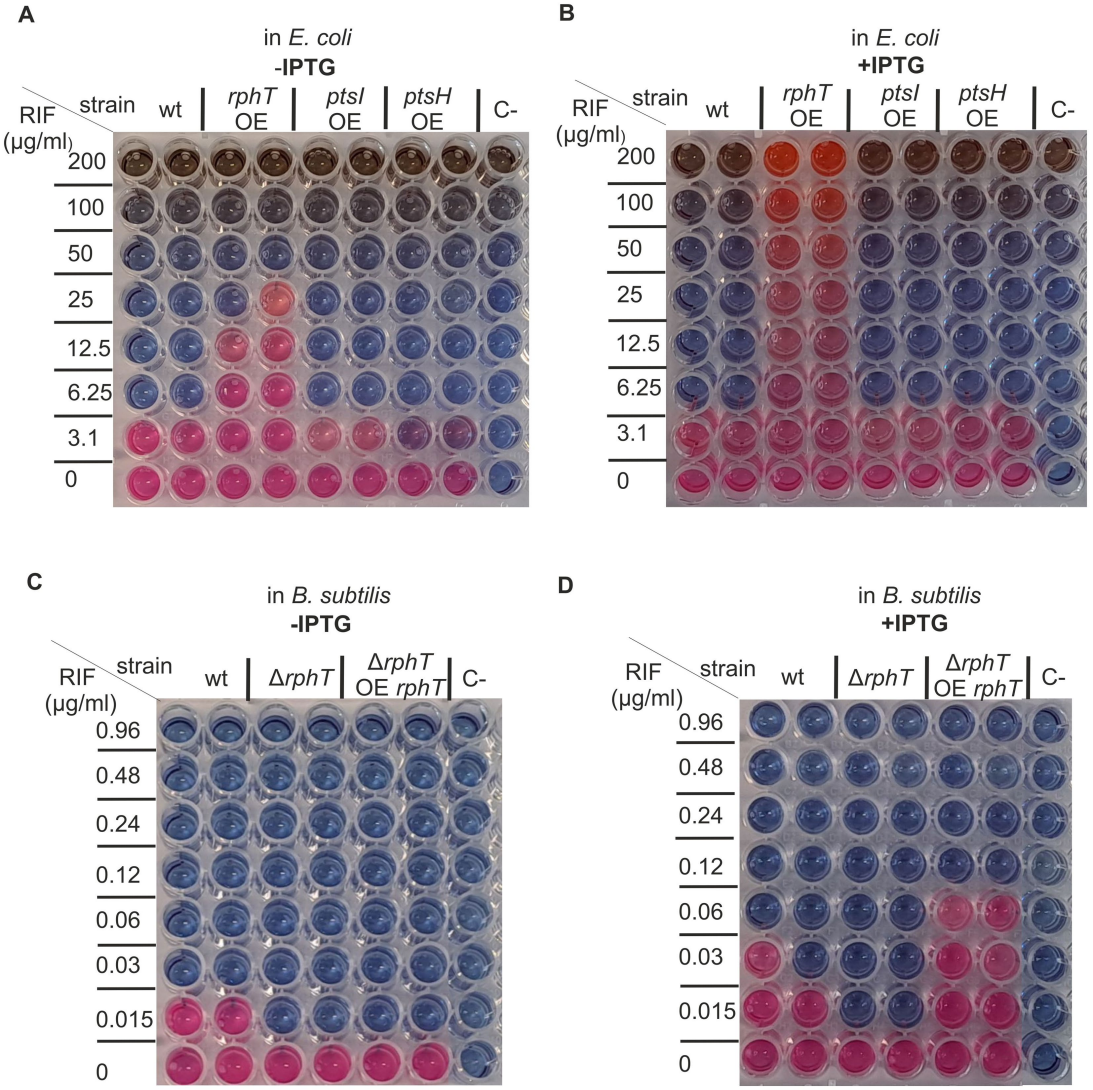
Overexpression of *B. subtilis rphT* increases rifampicin MIC in *E. coli* and in *B. subtilis*. (**A, B)** MIC of *E. coli* strains overexpressing *B. subtilis rphT* (=RphT protein), *ptsI* (=EI protein), and *ptsH* (=HPr protein) from an inducible promoter in the absence **(A)** and presence **(B)** of 0.5 mM IPTG inducer. (**C, D)** MIC of *B. subtilis* wild type (WT), *ΔrphT* (=RphT protein), and *rphT* (=RphT protein) overexpressing strain (=*rphT* OE *ΔrphT*) in the absence **(C)** and presence **(D)** of 0.5 mM IPTG inducer. C- negative control, no bacterial culture was added.

To examine the potential of RphT to provide strong protection against rifampicin, an IPTG-inducible *rphT* construct was introduced into a heterologous host, *Escherichia coli*. The same types of constructs/strains bearing inducible *ptsI* or *ptsH*, respectively, were generated in parallel. While the latter two constructs had no detectable effect on the viability of *E. coli* cells in the presence of increasing rifampicin concentration, overexpression of *RphT* raised the MIC from 6 μg/mL (wt) to >200 μg/mL, the highest concentration of the antibiotic that was possible to test due to solubility constraints (Figures 8A,B). In *B. subtilis*, IPTG induction of rphT then allowed growth at rifampicin concentrations roughly twice those tolerated by the parental strain (Figures 8C,D)—likely due to gene dosage effects because in *E. coli* the construct is on a multicopy plasmid while in *B. subtilis* it integrates in a single copy at the *amyE* locus. Furthermore, rifampicin flux to the cell *via* free diffusion is easier in Gram-positive bacteria than in Gram-negative bacteria, owing to differences in cell wall composition. This property may also play a role in the different rifampicin susceptibility.

These results reveal that, if strongly expressed even in a heterologous host, the *rph* gene can generate a “super-resistant” phenotype.

## 4 Discussion

Studying how antibiotic resistance emerged and spread in the past is the key to predicting its future: deciphering the origins and evolution of antibiotic resistance may help to anticipate how resistance to new antibiotics will arise. In this study, we have uncovered a hierarchy and potential evolutionary links between rifampicin tolerance mechanisms in *B. subtilis*: (1) RphT is a dedicated, high-efficiency rifampicin phosphotransferase that confers strong protection when expressed at high levels, (2) PTS proteins EI, HPr and probably MtlF increase fitness in the presence of subinhibitory rifampicin concentration, indicating that the intact PTS cascade supports basal tolerance (likely through regulatory networks rather than direct drug modification), (3) EI, a structural homolog of an RPH domain, displays residual rifampicin phosphorylating activity *in vitro* but this activity likely does not contribute to rifampicin resistance *in vivo*.

These results are in accord with a model in which the multi-domain RPH enzyme arose from a fusion of ancestral PTS components, gaining a specialized and highly efficient rifampicin-inactivating function.

The RPH enzyme has been found in many environmental and pathogenic bacteria, including Actinomycetes, Nocardia, and the human pathogen *Listeria monocytogenes*. *Bacillus* strains were known to be able to inactivate rifampicin by phosphorylation (Dabbs et al., 1995) and a RPH homologue, RphT (62% AA identity with *L. monocytogenes* RPH), was found in the genome. This specialized *B. subtilis* RphT enzyme encoded by *pps* (= *rphT*) is highly efficient and, like other RPHs, inactivates rifampicin by phosphorylation.

Among the PTS proteins, only EI exhibits ATP-dependent rifampicin phosphorylating activity *in vitro*. This activity is approximately two orders of magnitude lower than that of RphT and is comparable to the activity observed for the whole PTS complex. Rifampicin phosphorylation by EI depends on residues S34 and S36 and, to a lesser extent, H189. Thus, it appears to depend on different amino acid residues than phosphorylation of its physiological target, HPr.

The exact mechanism of rifampicin phosphorylation is currently unclear. EI, however, is a highly dynamic protein, and so this process may involve local and global conformational rearrangements, including a monomer-to-dimer transition (Nguyen et al., 2021; Sedinkin et al., 2024) that was shown to be important for autophosphorylation of EI (Lee et al., 2017). The rifampicin phosphorylating activity, however, could not be detected *in vivo* even though EI was shown to phase separate and locally reach relatively high concentrations, at least in *E. coli* (Albocher-Kedem et al., 2025). Yet, EI and HPr contribute to rifampicin resistance as demonstrated in the current study, and these two proteins were recently also shown to be important for virulence of *L. monocytogenes* (Freeman et al., 2025). These effects could be due to the importance of these proteins for carbon metabolism and, consequently, the fitness of the cell. Alternatively, the role of PTS proteins in rifampicin resistance could be explained by the involvement of HPr, probably together with EI, in gene expression regulation via the transcription factor CcpA (Deutscher et al., 1995), without directly modifying the drug. However, the precise role of PTS proteins in antibiotic resistance remains largely unclear. The regulatory role of these proteins in rifampicin resistance is being studied in our laboratory, and the results will be published in due time.

Taken together, our findings provide the first biochemical evidence that the PTS component possesses a promiscuous rifampicin-phosphorylating activity, consistent with the hypothesis that RPH evolved from a PTS ancestor (Stogios et al., 2016). A possible evolutionary scenario might be that an ancestral EI protein possessed a weak ATP-dependent ability to phosphorylate chemically diverse hydroxyl-carrying molecules, including rifampicin. Through gene duplication and fusion events, the HPr, EI, EIIA, and PEPs family proteins were fused to form a multidomain scaffold that could position rifampicin optimally for phosphorylation. Finally, adaptive optimization, including mutations enhancing substrate affinity, improving catalytic activity, and releasing the modified molecule, yielded the modern, highly effective RPH enzyme. This hypothesis is supported by examples from the literature about the evolution of multidomain proteins by domain recruitment, fusion and shuffling (Basu et al., 2009; Marsh and Teichmann, 2010). Genome sequencing projects have also uncovered an intriguing group of chimeras that contain homologous domains to either one or both general PTS proteins (HPr, EI), often combined with sugar-specific EII complexes (Hu and Saier, 2002; Deutscher et al., 2006). An example is the *M. smegmatis* PtsT protein of unknown function that comprises domains homologous to EIIA, HPr, and EI5 (Titgemeyer et al., 2007) or *E. coli* DhaM, composed of a mannose-like EIIA domain followed by an HPr and a truncated EI domain (Gutknecht et al., 2001). *B. subtilis* genome encodes also another structural homolog of RphT, a flavonoid phosphate synthetase YvkC, which phosphorylates a broad spectrum of flavonoids (Hsu et al., 2023). Additionally, improvement of substrate specificity and catalytic activity during evolution can be exemplified by the evolution of metallo-β-lactamase or actinobacterial PTS enzymes (Baier et al., 2019; Yang et al., 2020).

Finally, this study reveals that the dedicated, efficient RPH proteins have the potential to confer high levels of rifampicin resistance. RPH homologs are widespread across many bacterial species that are phenotypically sensitive to rifampicin, such as the human pathogen *Listeria monocytogenes* or *B. subtilis*, where the *rphT* gene is expressed at relatively low basal levels (Spanogiannopoulos et al., 2014). This level can be increased by more than an order of magnitude by the presence of subinhibitory amounts of rifampicin, providing moderate resistance. This level of resistance can be further increased, as shown in *B. subtilis*, where overexpression of a single copy of the *rphT* gene raised the rifampicin MIC by two-fold. More alarmingly, expression of *rphT* from a plasmid in *E. coli* produced resistance even to the highest rifampicin concentration tested (>200 μg/mL). These observations suggest that a latent, high-potency resistance potential exists in diverse bacteria and can be unleashed by promoter activation or horizontal gene transfer, as previously documented, e.g., for *β*-lactamases or sulfonamides (Cazares et al., 2025; Kaul et al., 2025).

## Data Availability

The datasets presented in this study can be found in online repositories. The names of the repository/repositories and accession number(s) can be found at: MassIVE: LC–MS primary data https://massive.ucsd.edu/ProteoSAFe/dataset.jsp?accession=MSV000100512 Molecular Biophysics Database: raw MicroScale Thermophoresis data https://doi.org/10.71479/gf2bm-df854.
