# Supplementary material for: The bacterial phosphotransferase system-mediated rifampicin phosphorylation: ancestral links to rifampicin-inactivating enzyme
Source: Front Microbiol. 2026 Apr 8;17:1789656. doi: 10.3389/fmicb.2026.1789656 (PMC13099321; doi:10.3389/fmicb.2026.1789656)
Supplement: Supplementary file 1 [file Data_Sheet_1.pdf]

Analysis Report

Analysis Name:	Analysis-Set #1
Type of Analysis:	MST
Evaluation strategy:	On Time
Cold Region Start:	-1 s
Cold Region End:	0 s
Hot Region Start:	1.5 s
Hot Region End:	2.5 s
Exported on:	Mon, 06 Oct 2025 10:36:52 GMT
Software Version:	MO.Affinity Analysis v2.2.4

Compare Kd-Fit: Analysis-Set #1

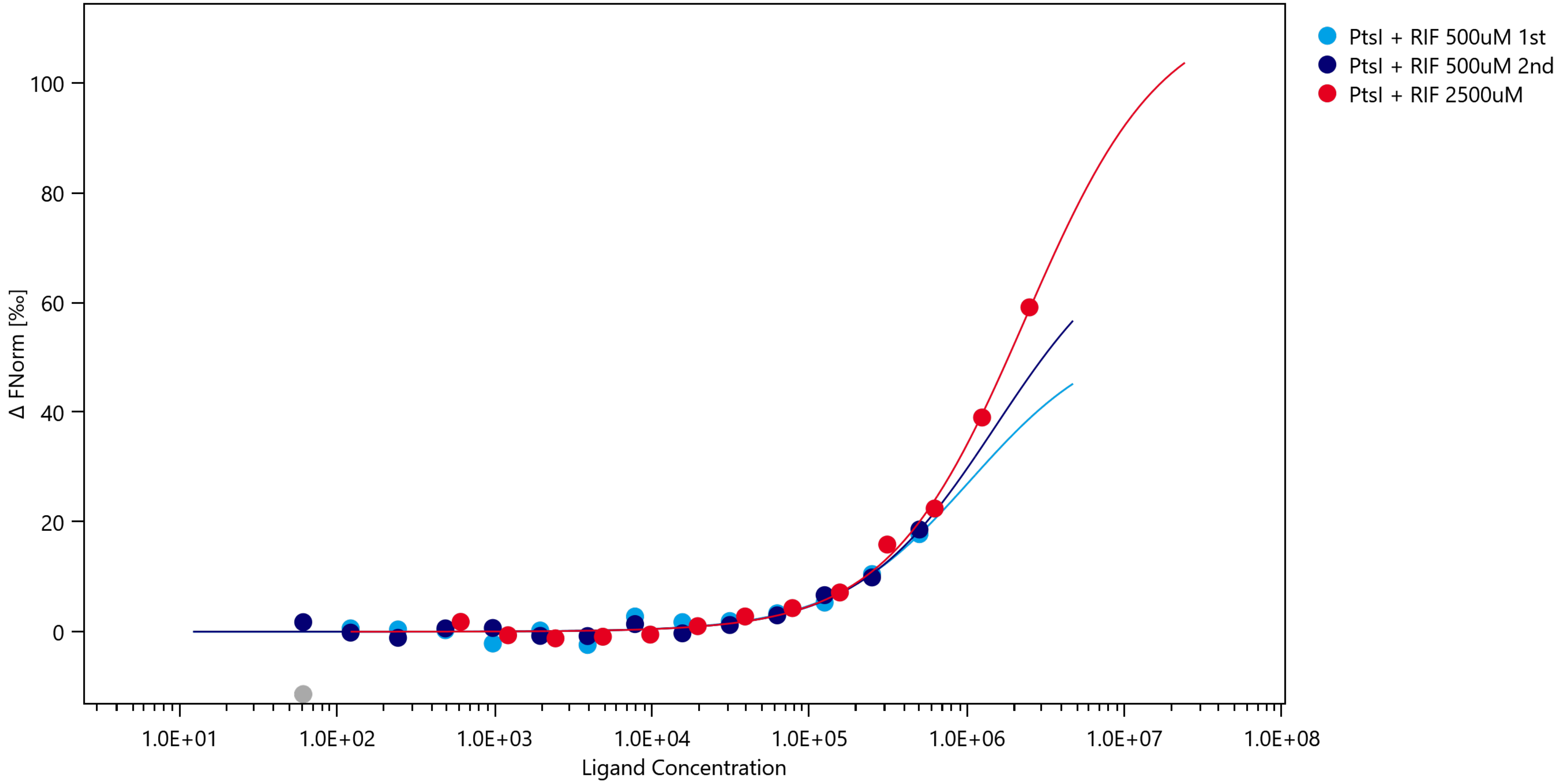

Dataset Overview

Name:	PtsI + RIF 500uM 1st	PtsI + RIF 500uM 2nd	PtsI + RIF 2500uM
Graph Color:	●	●	●
Target Name:	Target	Target	Target
Target Concentration:	100	100	100
Ligand Name:	Ligand	Ligand	Ligand
Ligand Concentration:	5E+05 to 122	5E+05 to 61	2.5E+06 to 610
n:	1	1	1
Comments:			
Excitation Power:	30%	30%	30%
MST Power:	50%	50%	50%
Temperature:	22.0°C	22.0°C	22.0°C
Kd:	1.0601E+06	1.5258E+06	2.3387E+06
Kd Confidence:	± 9.2353E+05	± 1.1572E+06	± 3.6229E+05
Response Amplitude:	55.32745	74.960754	113.67244
TargetConc:	100[Fixed]	100[Fixed]	100[Fixed]
Unbound:	916.92	903.82	903.06
Bound:	972.25	978.78	1016.7
Std. Error of Regression:	1.385604	0.97139171	1.3372304
Reduced χ^2:			
Signal to Noise:	43.741346	83.890907	93.119273

Analysis-Set #1 - MST-Traces

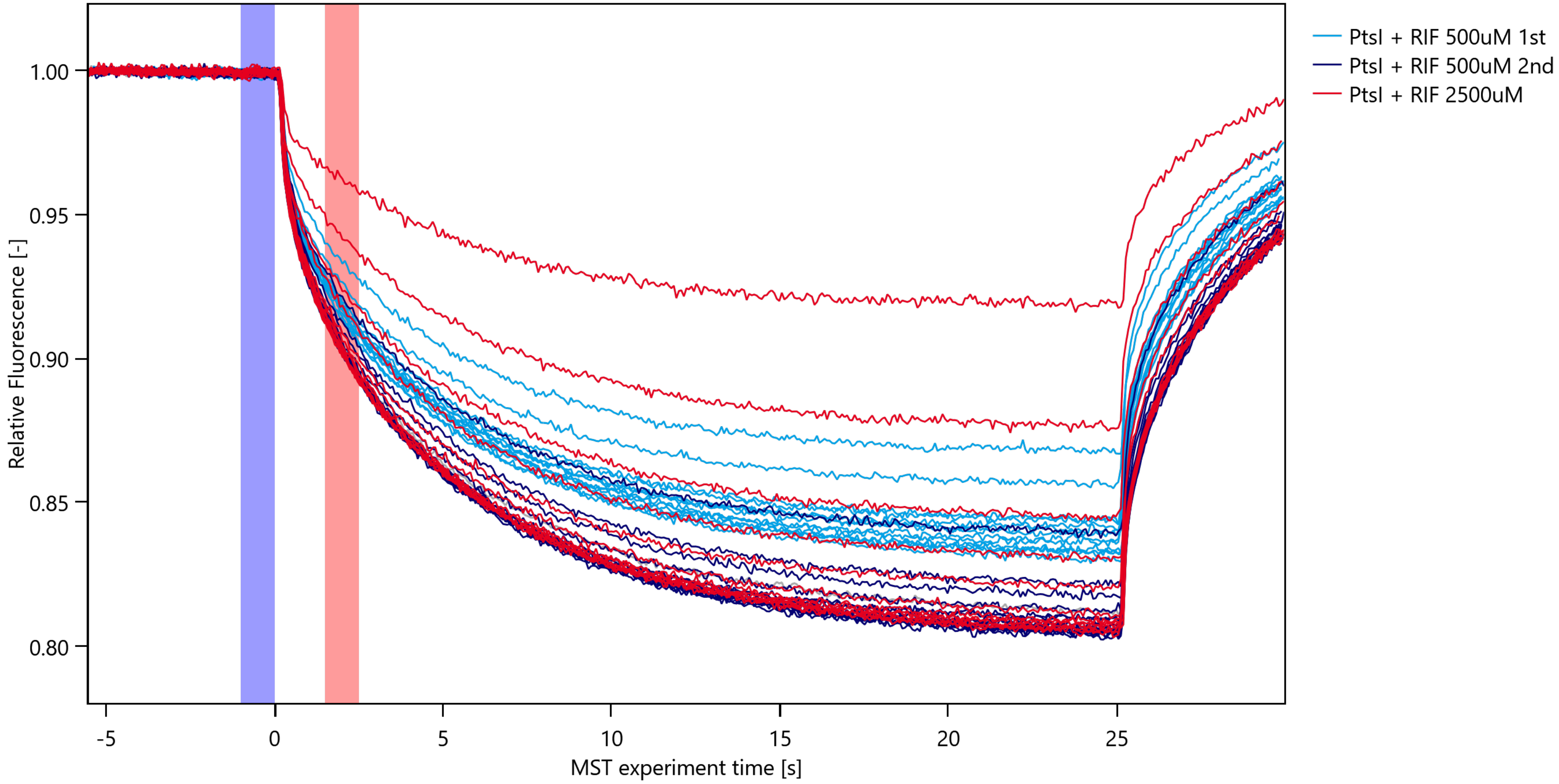

Analysis-Set #1 - Capillary Scan

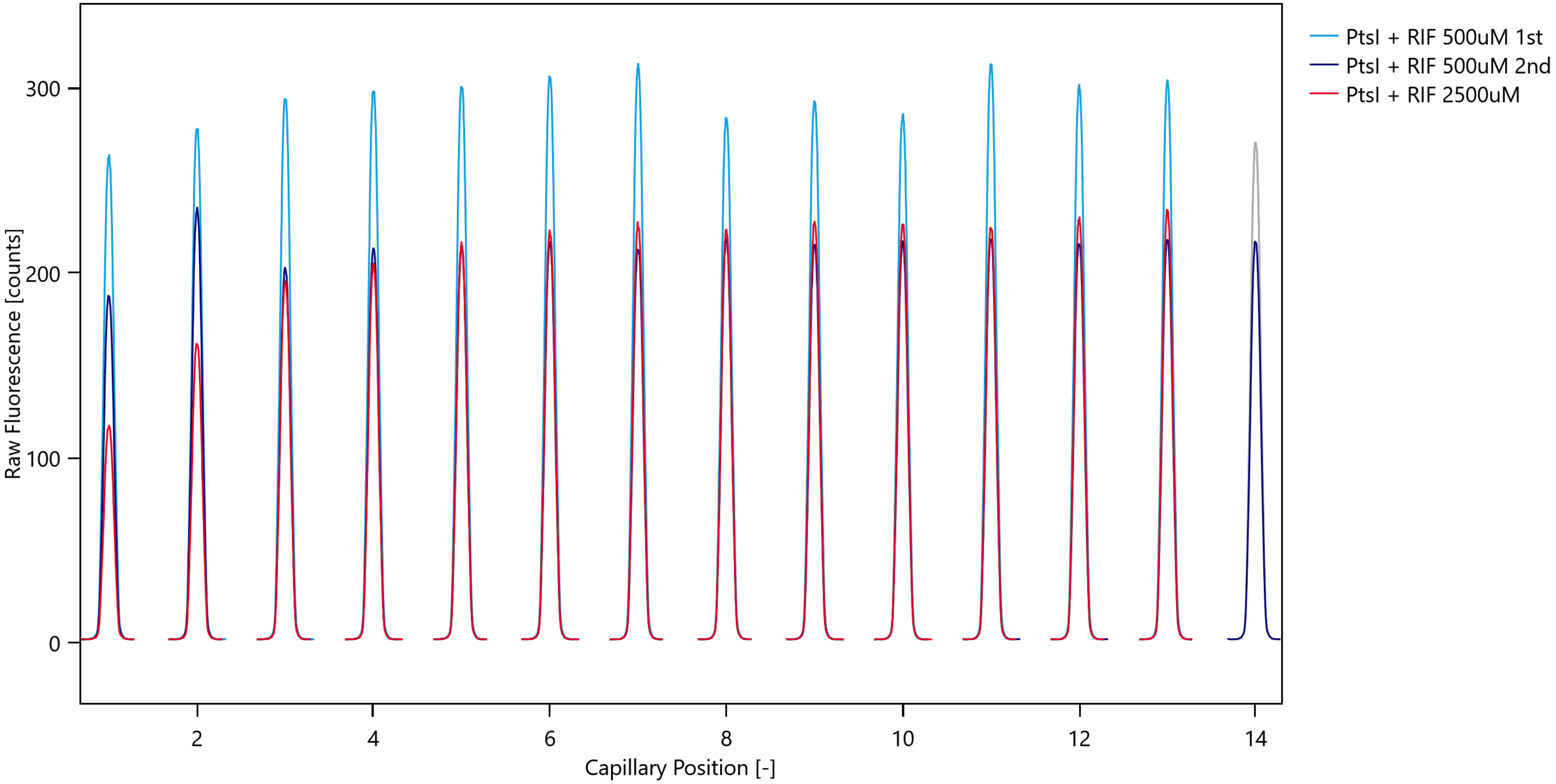

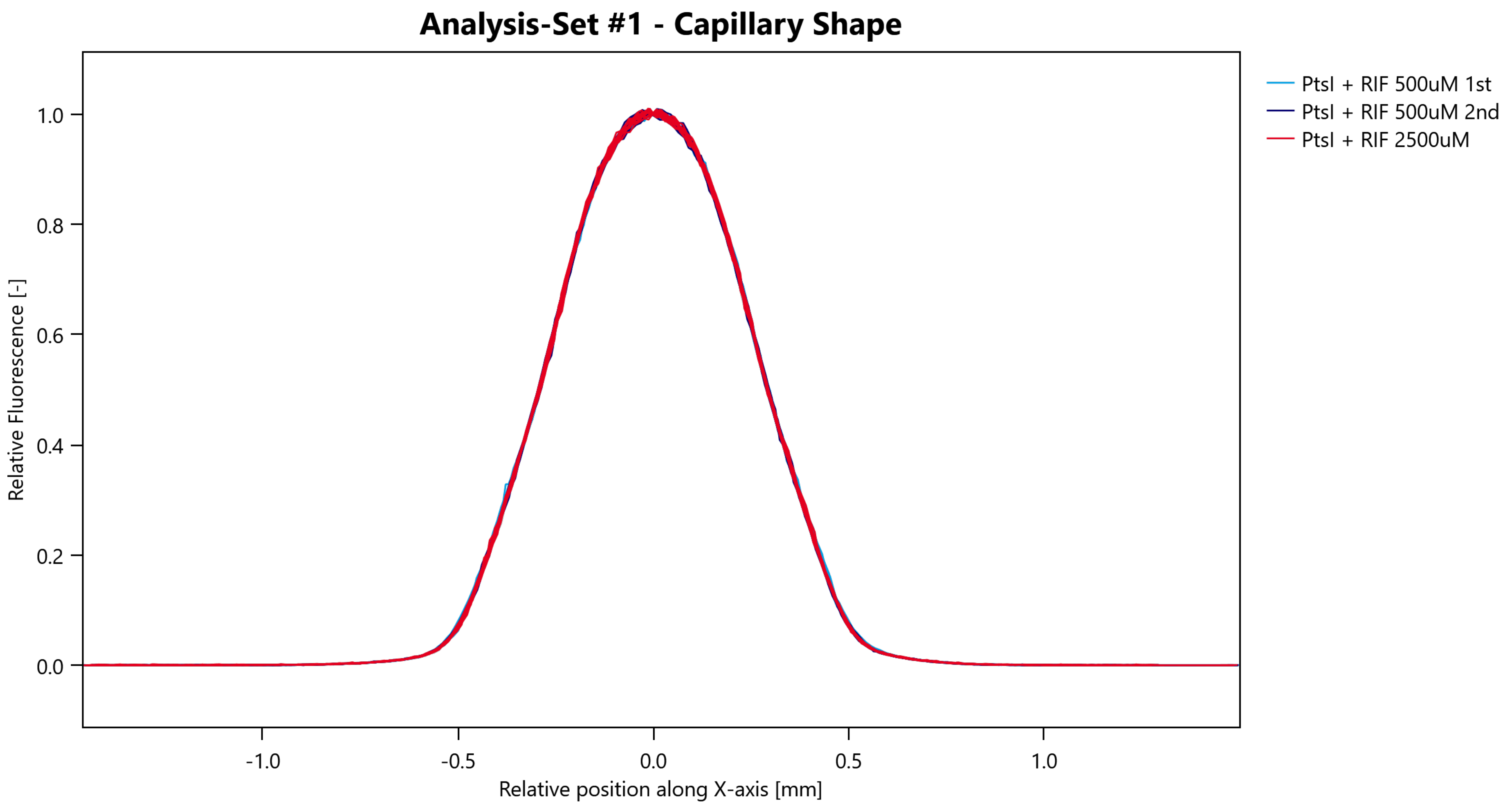
Analysis-Set #1 - Capillary Shape
Relative Fluorescence [-]
1.0
0.8
0.6
0.4
0.2
0.0
-1.0
-0.5
0.0
0.5
1.0
Relative position along X-axis [mm]
PtsI + RIF 500uM 1st
PtsI + RIF 500uM 2nd
PtsI + RIF 2500uM

Raw data of merged Dose-Response #1
PtsI + RIF 500uM 1st

Dose	Response (Average)	Std. Dev.	n
500000	934.71694		1
250000	927.42403		1
125000	922.23913		1
62500	920.28212		1
31250	918.86671		1
15625	918.66842		1
7812.5	919.69635		1
3906.25	914.51864		1
1953.125	917.14335		1
976.5625	914.77871		1
488.2813	917.20556		1
244.1406	917.33368		1
122.0703	917.53057		1

Outlier

Dose	Response	Std. Dev.	Number of merged Points
61.0352	905.53665		0

Raw data of merged Dose-Response #2
PtsI + RIF 500uM 2nd

Dose	Response (Average)	Std. Dev.	n
500000	922.44241		1
250000	913.713		1
125000	910.48463		1
62500	906.80613		1
31250	905.04224		1
15625	903.52584		1
7812.5	905.20593		1
3906.25	903.02944		1
1953.125	903.06134		1
976.5625	904.50022		1
488.2813	904.42596		1
244.1406	902.70653		1
122.0703	903.65469		1
61.0352	905.56172		1

Raw data of merged Dose-Response #3
PtsI + RIF 2500uM

Dose	Response (Average)	Std. Dev.	n
2500000	962.16864		1
1250000	942.09347		1
625000	925.50789		1
312500	918.94746		1
156250	910.21238		1
78125	907.36186		1
39062.5	905.83898		1
19531.25	904.0744		1
9765.625	902.53584		1
4882.8125	902.14706		1
2441.4063	901.85652		1
1220.7031	902.42027		1
610.3516	904.85762		1
