# Supplementary material for: The bacterial phosphotransferase system-mediated rifampicin phosphorylation: ancestral links to rifampicin-inactivating enzyme
Source: Front Microbiol. 2026 Apr 8;17:1789656. doi: 10.3389/fmicb.2026.1789656 (PMC13099321; doi:10.3389/fmicb.2026.1789656)
Supplement: Supplementary file 2 [file Supplementary_File_1.docx]

***SUPPLEMENTARY MATERIAL***

**
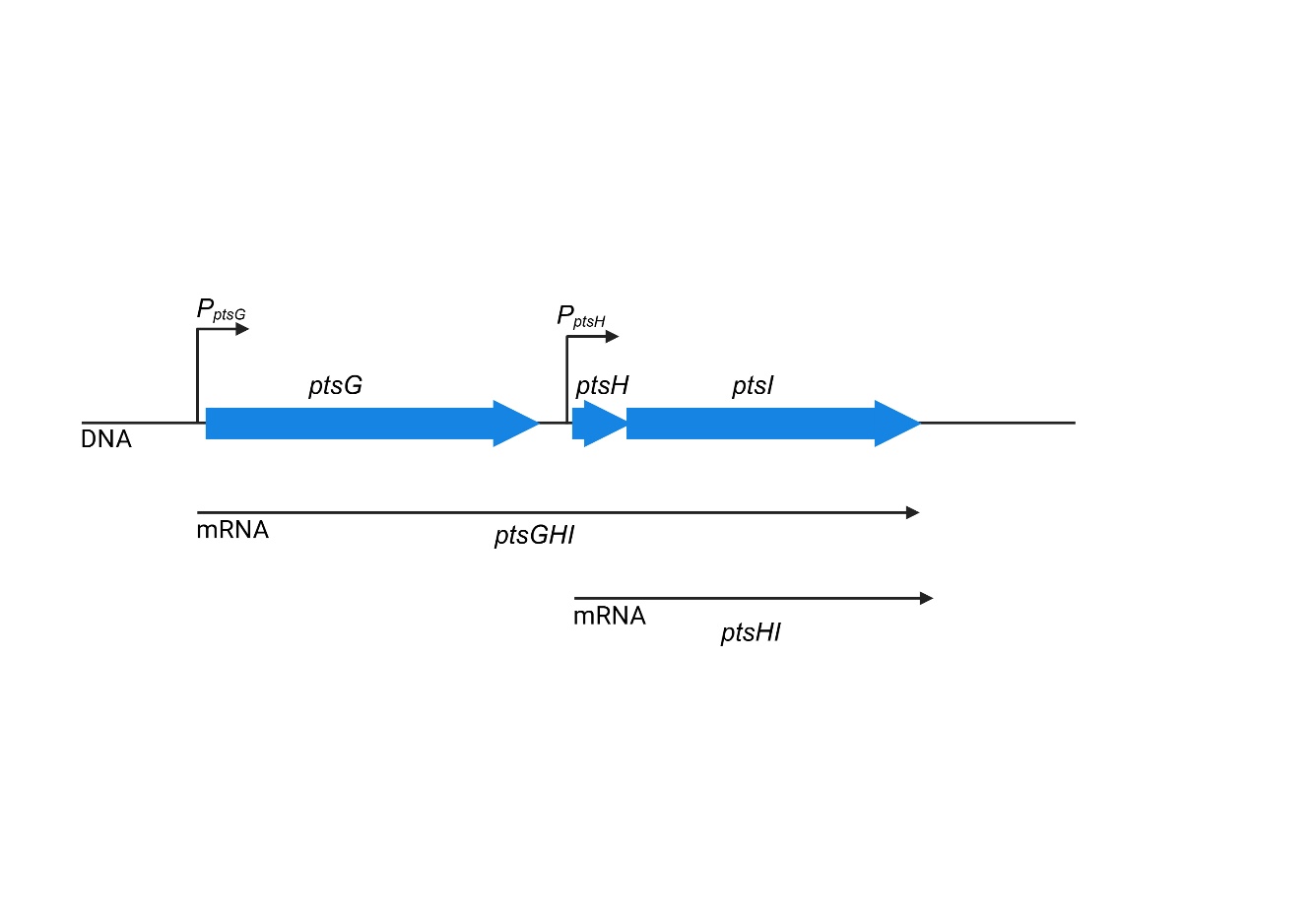
**

**Supplementary Figure 1 Organisation of the *ptsGHI* operon in *B. subtilis*.** In this region there are two promoters: P_ptsG_ for *ptsGHI* transcription and P_ptsH_ for *ptsHI* transcription (Stülke et al. 1997). Each of the promoters is independent.

**Supplementary Figure 2 Growth of *B. subtilis* *wt*, and *ptsI* deletion strain, and the two types of complementation strains (Δ*ptsI* complemented by *ptsHI* and Δ*ptsI* complemented by *ptsGHI*) in the absence and presence of subinhibitory concentrations of rifampicin (0.03 µg/ml). (A)** Growth without rifampicin **(B)** growth in the presence of rifampicin. The growth was performed in liquid LB medium in 96-well plates as described in Materials and Methods; averages of three biological repetitions are presented, and standard deviations from three independent experiments are depicted as shadow areas. Com = complementation

**
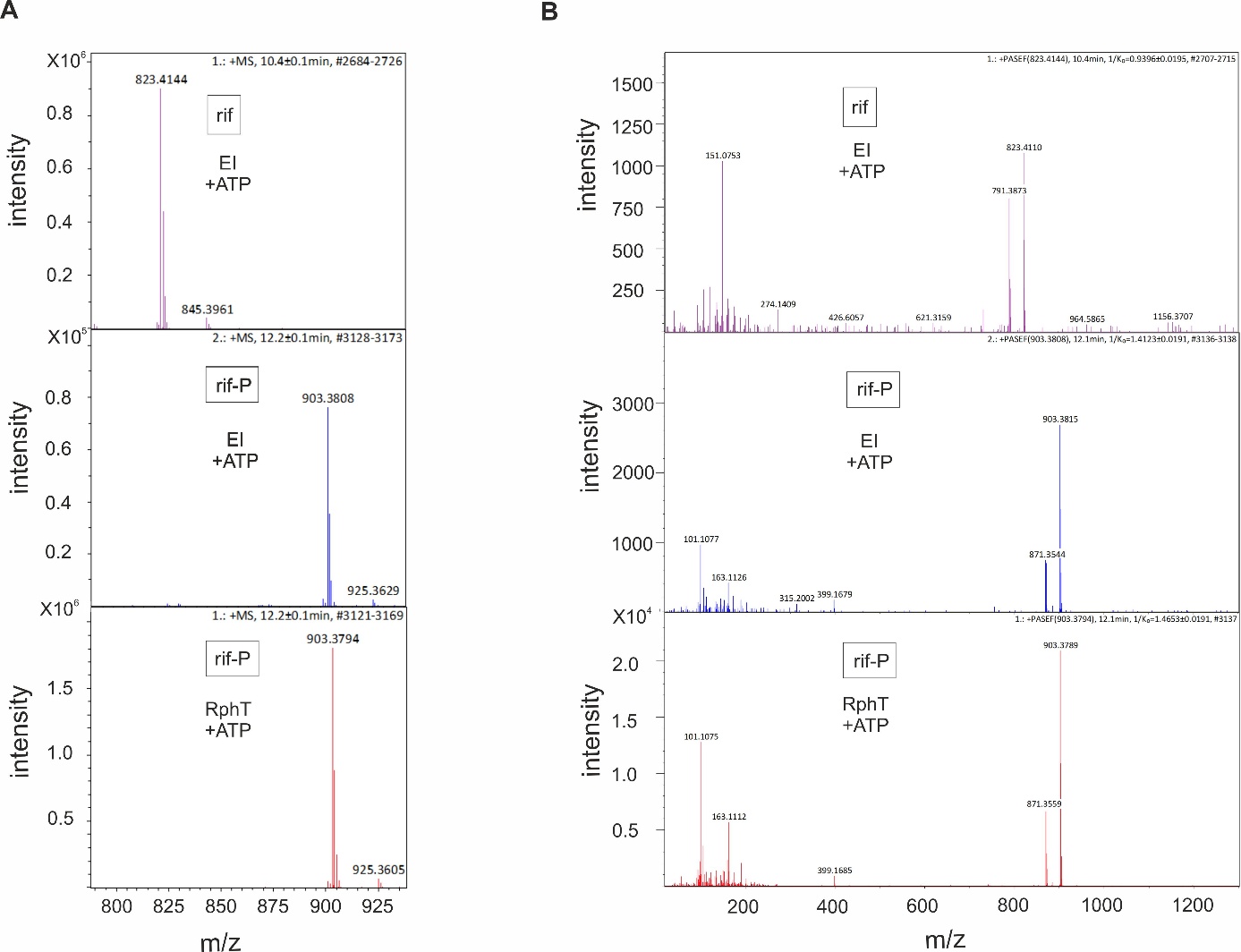
**

**Supplementary Figure 3 (A). High-resolution MS spectra of rifampicin (rif) and phosphorylated rifampicin (rif-P) detected in EI- and RphT-catalyzed *in vitro* reactions.** The theoretical [M+H]⁺ values are 823.41295 for rif and 903.37928 for rif-P. These correspond to mass errors of 1.76 ppm (rif) and 1.68 ppm (rif-P) in the EI-catalyzed reaction, and 0.13 ppm for rif-P in the RphT-catalyzed reaction. All observed mass errors fall within the specifications of the instrumentation. **(B). Collision-induced dissociation (CID) MS/MS spectra of rifampicin (rif) and phosphorylated rifampicin (rif-P) detected in EI- and RphT-catalyzed *in vitro* reactions.** The MS/MS spectra of both rif and rif-P exhibit a characteristic fragment ion corresponding to loss of a methoxy group.

**
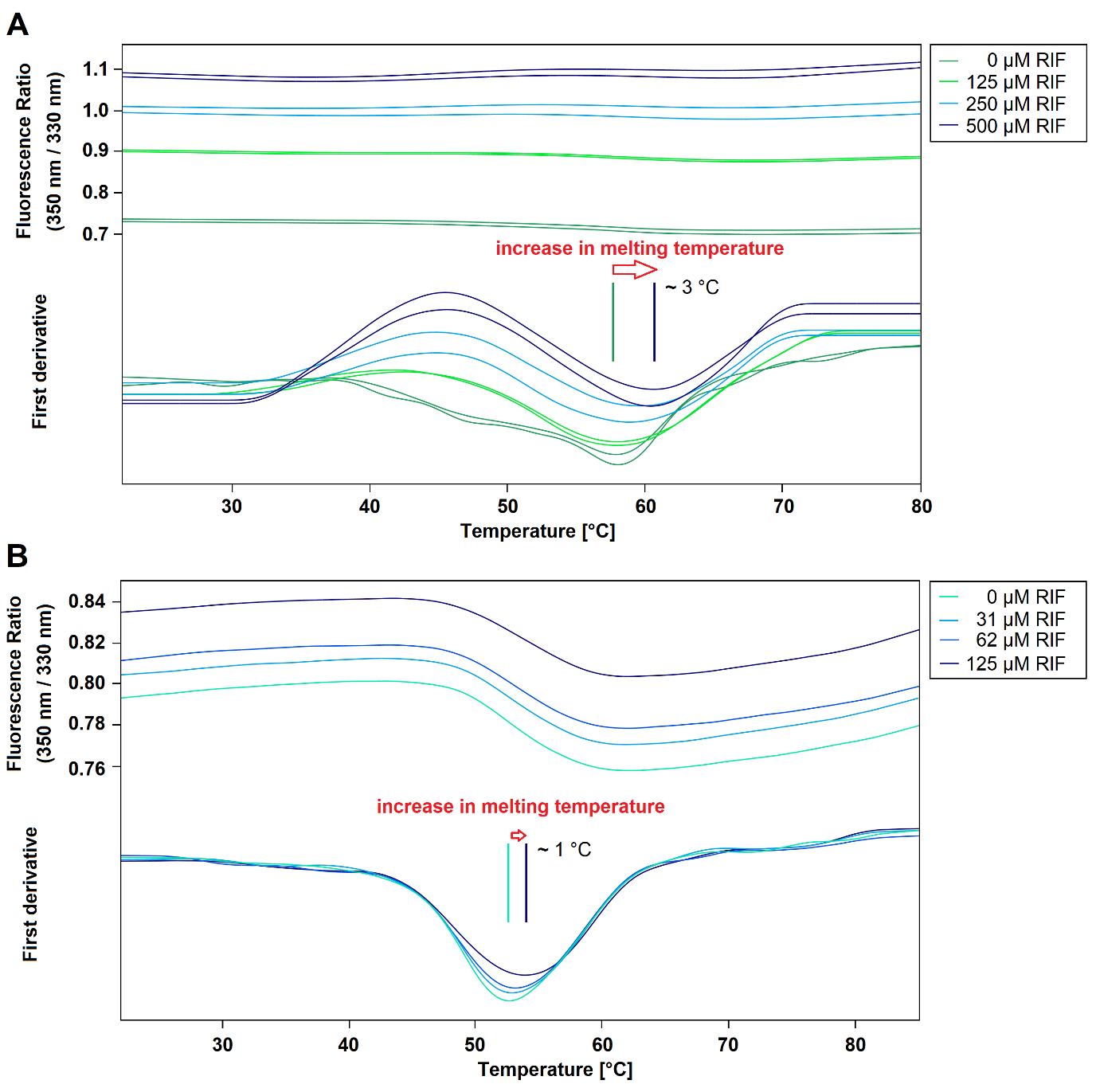
**

**Supplementary Figure 4 Interaction of EI and rifampicin analyzed using nano Differential Scanning Fluorimetry (nanoDSF).** Temperature conformational stability of EI in the presence of increasing concentration of rifampicin was measured using two different independently expressed and purified batches of EI in two different buffer systems: (**A)** 50 mM Bis-Tris pH 6.5 with 50 mM NaCl and (**B)** 40 mM Tris-HCl pH 8.0, 50 mM KCl, and 10 mM MgCl_2_. EI was stabilized by the presence of rifampicin in both cases. This is apparent from the increase in El conformational stability (marked by red arrow) in both titration series. All measurements were done using a Prometheus NT.48 and Prometheus Standard Capillaries (NanoTemper Technologies). The final concentration of EI in each measurement was 0.5 mg/ml. In (**A),** the measurement was done in a temperature range from 22 °C to 80 °C, using a 1.5 °C/min gradient and 100% excitation. Measurement was done in duplicates. In (**B),** the temperature range was 22 °C to 85 °C, gradient 1.5 °C/min, and excitation 90%. Data was measured, processed, and figures were created using the PR.ThermControl software v2.11 (NanoTemper Technologies).

**Supplementary Table 1.**

**Calculated doubling times (t_D_) of deletion strains used in this study.** The calculation was performed using the software available at: <https://dashing-growth-curves.ethz.ch/>. For statistical comparison one-way Anova test was used. Average values of doubling times from three independent biological replicates were compared with wild-type.

** p-value p < 0.01 **** p < 0.0001, ND = not determined

| **strain** | **doubling time [h] average and standard deviation** | **statistical significance** |
| --- | --- | --- |
| wt | 1.1 ± 0.12 | reference |
| wtRIF | 1.0 ± 0.08 | ns |
| Δ*rphT* | 1.2 ± 0.27 | ns |
| Δ*rphT*RIF | ND | ND |
| Δ*ptsH* | 1.7 ± 0.15 | ** |
| Δ*ptsH*RIF | 2.2 ± 0.39 | **** |
| Δ*ptsI* | 1.4 ± 0.10 | ns |
| Δ*ptsI*RIF | 1.7 ± 0.15 | ** |
| Δ*pckA* | 0.8 ± 0.19 | ns |
| Δ*pckA*RIF | 0.9 ± 0.16 | ns |
| Δ*mtlF* | 1.1 ± 0.14 | ns |
| Δ*mtlF*RIF | 1.0 ± 0.07 | ns |
